# The association between bromodomain proteins and cancer stemness in different solid tumor types

**DOI:** 10.1002/ijc.33937

**Published:** 2022-01-29

**Authors:** Patrycja Czerwinska, Anna Maria Jaworska, Nikola Agata Wlodarczyk, Małgorzata Cisek, Marianna Karwacka, Julia Lipowicz, Julia Ostapowicz, Monika Rosochowicz, Andrzej Adam Mackiewicz

**Affiliations:** ^1^ Department of Cancer Immunology Poznan University of Medical Sciences Poznan Poland; ^2^ Department of Diagnostics and Cancer Immunology Greater Poland Cancer Centre Poznan Poland

**Keywords:** ATAD2, BrD, bromodomain, bromodomain protein, cancer stemness, mRNA‐SI, TCGA

## Abstract

Cancer stemness, which covers the stem cell‐like molecular traits of cancer cells, is essential for tumor development, progression and relapse. Both transcriptional and epigenetic aberrations are essentially connected with cancer stemness. The engagement of bromodomain (BrD) proteins—a family of epigenetic factors—has been presented in the pathogenesis of several tumor types, although their association with cancer stemness remains largely unknown. Here, we harnessed TCGA and GEO databases and used several bioinformatic tools (ie, Oncomine, PrognoScan, GEPIA2, TIMER2.0, TISIDB, GSEA, R2 platform) to characterize the association between the BrD family members' expression and cancer stemness in solid tumors. Our results demonstrate that significant upregulation of *ATAD2* and *SMARCA4*, and downregulation of *SMARCA2* is consistently associated with enriched cancer stem cell‐like phenotype, respectively. Especially, higher‐grade tumors that display stem cell‐like properties overexpress *ATAD2*. In contrast to most BrD members, the gene expression profiles of *ATAD2* ^HIGH^ expressing tumors are strongly enriched with known markers of stem cells and with specific targets for c‐Myc transcription factor. For other BrD proteins, the association with cancer de‐differentiation status is rather tumor‐specific. Our results demonstrate for the first time the relation between distinct BrD family proteins and cancer stemness across 27 solid tumor types. Specifically, our approach allowed us to discover a robust association of high *ATAD2* expression with cancer stemness and reveal its' versatility in tumors. As bromodomains are attractive targets from a chemical and structural perspective, we propose *ATAD2* as a novel druggable target for de‐differentiated tumors, especially those overexpressing *MYC*.

## Graphical abstract

What's new?

While epigenetic alterations are linked to stem cell‐like molecular traits in cancer cells, associations between cancer stemness and aberrations in epigenetic factors known as bromodomain (BrD) proteins remain unclear. In the present investigation of associations between BrD protein expression and cancer progression and de‐differentiation status, several BrD members were linked to cancer stem cell‐like phenotypes in solid tumors. Notably, BrD family member *ATAD2* was found to be consistently overexpressed in high‐grade tumors with stem cell properties. The findings shed light on the molecular basis of de‐differentiation and could inform novel therapeutic strategies for eradicating tumors with stem cell‐like properties.

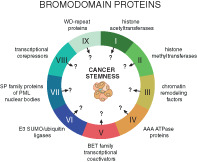

**Abbreviations**
ACC: adrenocortical carcinoma
BLCA: bladder urothelial carcinoma
BRCA: breast invasive carcinoma
BrD: bromodomain
CESC: cervical squamous cell carcinoma and endocervical adenocarcinoma
COAD: colorectal adenocarcinoma
CSC: cancer stem cell
DEG: differentially expressed genes
ESCA: esophageal carcinoma
FDR: false discovery rate
GBM: glioblastoma multiforme
GEO: gene expression omnibus
GSEA: gene set enrichment analysis
HNSC: head and neck squamous cell carcinoma
HPA: The Human Protein Atlas
HR: hazard ratio
IHC: immunohistochemistry
KICH: kidney chromophobe
KIRC: kidney renal clear cell carcinoma
KIRP: kidney renal papillary cell carcinoma
LGG: brain lower grade glioma
LIHC: liver hepatocellular carcinoma
LUAD: lung adenocarcinoma
LUSC: lung squamous cell carcinoma
MESO: mesothelioma
mRNA‐SI: mRNA stemness index
OS: overall survival
OV: ovarian serous cystadenocarcinoma
PAAD: pancreatic adenocarcinoma
PRAD: prostate adenocarcinoma
RSEM: RNA‐seq by expectation maximization
SARC: sarcoma
SKCM: skin cutaneous melanoma
STAD: stomach adenocarcinoma
TCGA: The Cancer Genome Atlas
TGCT: testicular germ cell tumor
THCA: thyroid carcinoma
THYM: thymoma
UCEC: uterine corpus endometrial carcinoma
UVM: uveal melanoma

## 1 INTRODUCTION

The bromodomain is an evolutionarily conserved, about 110 amino acid‐long, protein‐protein interaction domains that facilitates the recognition of acetylated lysine residues. This essential activity provides versatile functions to bromodomain‐containing (BrD) proteins, primarily associated with the chromatin‐templated gene transcription, recombination, replication and repair of the DNA.^1^ As the majority of BrD proteins also contain additional structurally conserved functional domains, they display diverse physiological activities, including posttranslational histone modifications (acetylation and methylation), chromatin remodeling and recruitment of distinct transcription factors. Moreover, BrD members affect both transcription initiation and elongation. Notably, all those functions are fundamental for epigenetic regulation of gene expression.^1^, ^2^

BrD family members can be classified into nine subgroups based on their major molecular functions.^1^ Group I of BrD family members comprises nine members of histone acetyltransferases, group II—2 proteins acting as histone methyltransferases, group III—11 members of chromatin remodeling factors, group IV—2 proteins with AAA ATPase activity, group V—4 members of BET transcriptional coactivators, group VI—4 proteins with E3 SUMO/ubiquitin ligase activities, group VII—4 SP family proteins of PML nuclear bodies, group VIII—2 transcriptional co‐repressors with MYND zinc‐finger domain and group IX—3 members of WD‐repeat proteins. However, all BrD members are epigenetic “readers,” and numerous of them are known to be involved in the pathogenesis of distinct human diseases, including cancer.^2^, ^3^, ^4^, ^5^

Epigenetic dysregulation of gene expression contributes to tumorigenicity (at least partially) via facilitating the self‐renewal of cancer cells.^6^ Cancer cells that possess the ability to self‐renew and to differentiate into the more specialized progeny are known as cancer stem cells (CSCs). This population exhibits highly metastatic potential and facilitates tumor relapse after treatment due to intrinsic resistance to standard therapies.^7^, ^8^ Moreover, the high plasticity of CSCs provides the transition between stem‐like and non‐stem‐like states. Therefore, it is difficult to unequivocally define the CSC population and to determine whether distinct tumor types are organized into a rigid hierarchy.^9^ An increasing number of data demonstrates that molecular features characteristic for stem cells (“stemness”) are indisputable for cancer progression and therapy resistance.^10^, ^11^ Although it is unclear whether the cancer stemness reflects the presence of bona fide CSCs, the molecular signatures sufficient for grading stem cell‐like phenotype essentially contribute to the development of novel therapeutic approaches that may directly target the stem cell‐like compartment of the tumor.^12^

Recent reports suggest that several BrD members play a role in the regulation of the cancer stem cell population in distinct types of tumors.^13^, ^14^, ^15^ Especially, the role of BRD4 protein, a member of BET transcriptional coactivators, has been well documented in several studies in vitro. BRD4 regulates the self‐renewal of glioma,^16^ medulloblastoma,^17^ prostate,^18^ breast^19^ and stomach cancer stem cells,^20^ and is essential for normal stem cell maintenance.^15^, ^21^ Similarly, TRIM28 (also known as TIF1‐β or KAP1) facilitates stemness acquisition in distinct types of tumors, including breast,^22^, ^23^ lung^24^ and melanoma,^25^ and contributes to the stemness machinery of normal stem cells on several distinct levels.^26^, ^27^

However, little is known about other BrD family members and their association with cancer stemness. Here, we harnessed publicly available transcriptomic data from 27 distinct types of TCGA tumors to delineate the connection between specific BrD family proteins and cancer stemness measured with previously reported stemness indices or signatures.^10^, ^11^, ^12^, ^28^ Using the TIMER2.0 platform,^29^ we reported that for most BrD family members, the differential expression in tumor tissues vs normal adjacent tissues could be observed. According to the GEPIA2 database,^30^ for several BrD genes, we demonstrated a significant correlation with TCGA cancer patients' outcomes, mostly negative. These results were further validated with additional datasets from the Oncomine,^31^ PrognoScan^32^ and the GEO databases. Next, we used a transcriptome‐based stemness index (mRNA‐SI) and other stem cell‐derived gene expression signatures^10^, ^11^, ^12^, ^28^ to analyze the relation between BrD proteins' expression and the level of tumor de‐differentiation. We observed that among 41 tested BrD family members, the correlation with cancer stemness across 27 tumor types was consistently positive or negative for only five and four BrD genes, respectively (namely *ATAD2*, *BRD7*, *KAT2A*, *SMARCA4*, *TRIM28* [positive], and *KAT2B*, *BAZ2B*, *SP100* and *SMARCA2* [negative]). Using clinicopathologic data, we demonstrated that higher‐grade tumors display significant upregulation of *ATAD2* and *SMARCA4* expression, and downregulation of *SMARCA2* expression, which further confirms a universal relation of these BrD proteins' expression with cancer stemness. Moreover, the gene set enrichment analysis (GSEA)^33^ revealed that ATAD2‐associated and SMARCA4‐associated transcriptome profiles are significantly enriched with known markers of stem cells. On the other hand, *SMARCA2* ^HIGH^ and *KAT2B* ^HIGH^ tumors are significantly depleted with stemness markers. Further GSEA analyses with MSigDB Hallmark (v7.4) gene sets as a reference confirmed robust enrichment of *ATAD2*‐associated transcriptome profiles with “cancer hallmark” terms specific for stemness‐high tumors, especially the activation of c‐Myc‐dependent transcription.

Altogether, our results clearly demonstrate yet unrecognized association of high *ATAD2* expression with cancer stem cell‐like phenotype of solid tumors, regardless of the tumor type. However, molecular studies are necessary to determine the precise role of *ATAD2* in stem cell‐like tumors and to ascertain whether epigenetic functions mediated by *ATAD2* are sufficient to promote cancer stemness.

## 2 MATERIALS AND METHODS

### 2.1 TCGA solid tumor types selected for the study

In the current study, we selected 27 solid TCGA tumor types (a) with more than 50 samples collected and (b) with survival data available (tumor types that were excluded: CHOL, PCPG and UCS) for analyses (Table S1). All data is available online, and the access is unrestricted and does not require patients' consent or other permissions. The use of the data does not violate the rights of any person or any institution.

### 2.2 The expression of BrD family members in distinct TCGA cohorts

The differential expression of BrD family members (Table S2) in tumor tissues vs normal adjacent tissues across 18 out of 27 tested solid TCGA tumor types was analyzed using the Gene_DE module of the TIMER2.0 platform (http://timer.cistrome.org/).^29^ Only those tumor types for which normal adjacent tissue was available were analyzed with TIMER2.0. The Gene_DE module allows users to study the differential expression (log2‐normalized TPM values) between tumor and adjacent normal tissues for any gene of interest across TCGA tumors. The statistical significance was computed by the Wilcoxon test.

The data regarding BrD family members' expression in other GEO datasets was retrieved from the online database, Oncomine (https://www.oncomine.org/resource/login.html).^30^ For further details, see Supporting Information Materials and Methods and Figure S1.

### 2.3 The association between BrD family members expression and patients' outcome

The association between BrD family members expression and patients' overall survival (OS) across 27 solid TCGA tumor types was analyzed with the Survival_Map panel of the GEPIA2 database (http://gepia2.cancer-pku.cn/#index).^31^ The hazard ratio was estimated using the Mantel–Cox test using the mean BrD family members' expression as a cut‐off. As for additional GEO cohorts, the PrognoScan (http://dna00.bio.kyutech.ac.jp/PrognoScan/)^32^ database was used for the meta‐analysis of the prognostic value of various genes. For further details, see Supporting Information Materials and Methods and Table S3.

### 2.4 TCGA genomic data

Genomic data for 27 solid TCGA tumors were directly downloaded from the cBioportal (www.cbioportal.org) database.^33^

### 2.5 Transcriptomic data

The RNA sequencing‐based mRNA expression data were directly downloaded from the cBioportal. RNASeq V2 from TCGA is processed and normalized using RSEM.^34^ Specifically, the RNASeq V2 data in cBioPortal corresponds to the rsem.genes.normalized_results file from TCGA. The Spearman's correlation was used for detection of co‐expressed genes with *P*‐value <.05 and FDR < 0.01 as cut‐offs. Differentially expressed genes (DEGs) were cut off at *P*‐value <.05 and FDR < 0.05.

### 2.6 Stemness‐associated scores

The mRNA‐SI stemness score^12^ and other stemness signatures (Ben‐Porath_ES_core, Wong_ESC_core and Bhattacharya_ESC) used in this study were previously described.^10^, ^11^, ^28^ Briefly, the mRNA‐SI signature was calculated based on previously built predictive model using one‐class logistic regression (OCLR) on the pluripotent stem cell samples (ESC and iPSC) from the PCBC dataset. The resulting training matrix contained 12 945 mRNA expression values measured across all available PCBC samples. The obtained signature was further applied to score TCGA samples using the Spearman correlations between the model's weight vector and the sample's expression profile. The index was subsequently mapped to the [0,1] range by using a linear transformation that subtracted the minimum and divided by the maximum. As for Ben‐Porath_ES_core,^10^ Wong_ESC_core^11^ and Bhattacharya_ESC^28^ signatures, we used R2 platform (http://r2.amc.nl, accessed on 25 June 2021) to calculate the mean value (log2‐transformed *z*‐score) for each of the signatures in tested samples (each TCGA tumor type separately).

### 2.7 Histologic tumor grades

The association between BrD family members' expression and the histologic tumor grade was assessed using the TISIDB portal (http://cis.hku.hk/TISIDB/index.php).^35^ The correlation was calculated using Spearman's rank correlation coefficient (*r*).

### 2.8 Gene set enrichment analysis

The GSEA (http://www.broad.mit.edu/gsea/index.html)^36^ was used to detect the coordinated expression of a priori defined groups of genes within the tested samples. Gene sets are available from the Molecular Signatures Database (MSigDB, http://www.broad.mit.edu/gsea/.msigdb/msigdb_index.html).^37^ All significantly DEGs were imported to GSEA. The GSEA was run according to the default parameters: each probe set was collapsed into a single gene vector (identified by its HUGO gene symbol), permutation number = 1000, and permutation type = “gene‐sets.” The FDR < 0.01 was used to correct for multiple comparisons and gene set sizes.

### 2.9 Validation datasets from the GEO database

Additional datasets used in this study (Table S4) for GSEA analyses were obtained from the R2 Genomics Analysis and Visualization Platform. All datasets were analyzed online using the R2 Platform (http://r2.amc.nl, accessed on 25 June 2021) to find genes that correlate with selected BrD family members' expression. All data are freely available online, and access is unrestricted and does not require patient consent or other permissions.

### 2.10 Statistical analyses

Statistical analyses were carried out with GraphPad Prism 8.0 software (GraphPad Software, Inc, La Jolla, California). Multiple comparisons were performed with the ANOVA test. The correlation between two variables was assessed with Spearman's rank correlation coefficient (*r*).

## 3 RESULTS

### 3.1 The expression of BrD family members in tumor and normal adjacent tissues and the association with cancer patients' survival

**FIGURE 1:**
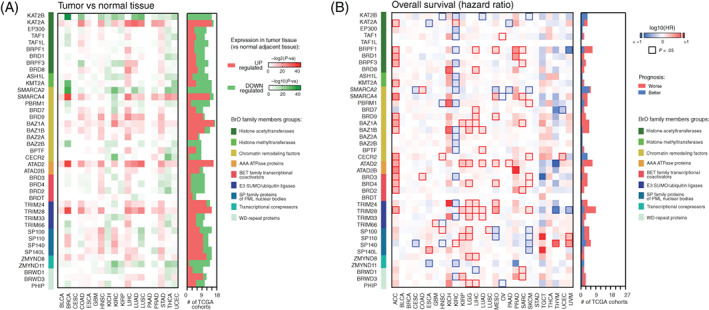
The expression of BrD family members in tumor and normal adjacent tissues and the association with cancer patients survival. (A) The differences in expression of BrD family members in tumor tissues and normal adjacent tissues based on TCGA data. The heatmap presents log10‐transformed statistical significance (*P*‐value). Color on the heatmap denotes either upregulated (red) or downregulated (green) expression in tumor tissues. TCGA tumor names are explained in Table S1. The number of TCGA cohorts characterized with either upregulated (red) or downregulated (green) expression of specific BrD family members in tumor tissues are shown. (B) The heatmap of the hazard ratio (log10[HR]) of death for patients with high expression of specific BrD family members (with the mean as a cut‐off). Red and blue denote higher or lower hazard ratios, respectively. Bordered squares denote statistically significant HRs. The number of TCGA cohorts characterized with either better (red) or worse (blue) prognosis are shown

Recent reports demonstrate that several BrD family members exhibit distinct expression patterns in tumor vs normal adjacent tissues. Here, we analyzed the expression for all 41 BrD family members (Tables S1 and S2) in 18 TCGA tumor types vs normal adjacent tissue using TIMER2.0 (http://timer.cistrome.org/).^29^ We observed that for most BrD family members, the differential expression highly depends on the tumor type. Only for five genes, namely *KAT2A*, *SMARCA4*, *BAZ1A*, *ATAD2* and *TRIM28*, we observed consistently higher levels and for two genes—*KAT2B* and *SMARCA2*—lower levels, respectively, regardless of the tumor type (Figure 1A). This observation was further validated with the data from the Oncomine database (Figure S1).^31^

Next, we used the average expression of BrD family members as a cut‐off for patients' stratification and observed that several BrD family members are substantially associated with a patient's outcome in TCGA datasets (Figure 1B). Especially in ACC and in LGG, the upregulation of 16 and 11 distinct BrD members, respectively, is associated with a worse prognosis. On the other hand, the upregulation of 18 distinct BrD family members in KIRC is associated with a better prognosis. As observed for the differential expression, the association of BrD family members with patient survival is tumor‐specific. For most BrD members, their level rarely correlates with tumor patients' survival, with only nine members (*BRPF1*, *SMARCA2*, *BAZ1A*, *BAZ1B*, *ATAD2*, *TRIM28*, *SP100*, *SP110* and *SP140*) being significantly associated with either better or worse survival of at least five distinct tumor types. We further validated this observation with additional datasets from the Prognoscan database (Figure S2).^32^

We also verified the frequencies of alterations in BrD family members with the cBioportal platform.^34^ As presented in Figure S3A, the mutation rates (including missense mutations, amplifications and deletions) in BrD member‐encoding genes were relatively low across all tested tumor types. Specifically, *ATAD2* exhibit the highest level of aberrations that accounts for 10% of all profiled samples (10 506 samples in 27 solid TCGA tumor types). A closer look at *ATAD2* mutation revealed a very high frequency of alterations in OV (35.3%) and elevated levels of alterations in ESCA (20.5%) and LIHC (20.2%; Figure S3B); most of these being amplifications.

### 3.2 The relation between BrD family members' expression and cancer stemness

**FIGURE 2:**
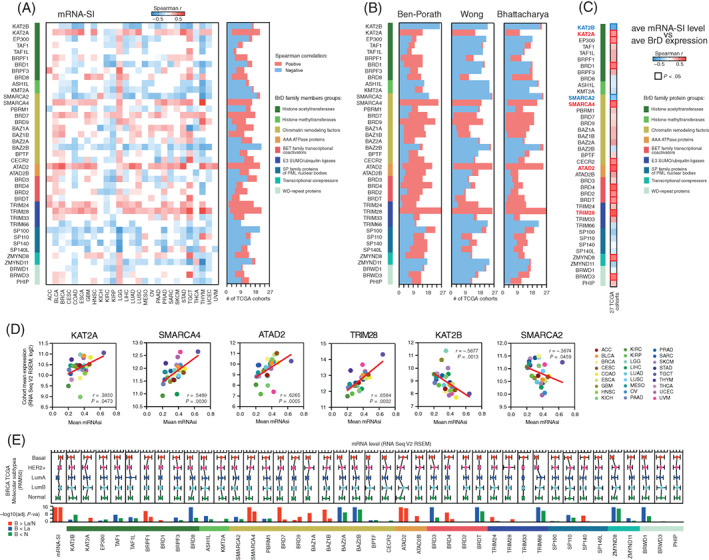
The association between BrD genes' expression and cancer stemness. (A) The heatmap of Spearman's correlation between BrD family members' expression and mRNA stemness index (mRNA‐SI) across 27 TCGA tumor types. The number of TCGA cohorts characterized with either a positive (red) or negative (blue) correlation between the expression of specific BrD family members and mRNA‐SI are shown. (B) The number of TCGA cohorts characterized with either positive (red) or negative (blue) correlation between the expression of specific BrD family members and tested stemness signatures (Ben‐Porath, Wong, Bhattacharya). (C) The association between the cohort mean mRNA‐SI level and the cohort mean expression (log2‐normalized) of BrD genes across 27 TCGA cohorts. (D) Dot plots of Spearman correlation between the cohort mean expression of selected BrD family members (*KAT2A*, *SMARCA4*, *TRIM28*, *ATAD2*, *KAT2B* and *SMARCA2*) and the cohort mean mRNA‐SI level. (E) The expression of BrD members in TCGA BRCA samples stratified by molecular subtypes (PAM50) into five subgroups: basal (red), HER2‐positive (magenta), luminal A (dark blue), luminal B (light blue) and normal‐like (green). The mean value with SD is plotted. Statistical significances (−log10[adj. *P*‐value]) of comparisons between basal vs luminal A and basal vs normal‐like BRCA subtypes calculated with Kruskal‐Wallis test followed by Dunn's multiple comparisons test are denoted below the graphs and color‐coded accordingly: red—BrD expression higher in basal vs luminal A or normal‐like (B > La/N); dark blue—BrD expression lower in basal vs luminal A (B < La); green—BrD expression lower in basal vs normal‐like subtype (B < N). For further details see Table S5

As previously reported, solid tumors display distinct levels of cancer stemness.^12^ Here, we analyzed the association between the expression of BrD family members and the level of tumor stemness quantified with the previously described transcriptome‐based stemness index (mRNA‐SI). As presented in Figure 2A, the expression of most BrD members significantly negatively correlated with cancer stemness across many TCGA tumor types, although only for *KAT2B*, *KMT2A*, *SMARCA2*, *BAZ2B*, *SP100* and *SP140*, the association was highly consistent (regardless of the tumor type). Moreover, we observed a significant positive correlation of *KAT2A*, *SMARCA4*, *ATAD2*, *TRIM24* and *TRIM28* expression with tumor stemness across most solid tumor types (with the last two members previously reported^38^). These associations were further confirmed with additional stem cell gene signatures (Figures 2B and S4), especially for *KAT2B*, *SMARCA2*, *SMARCA4*, *ATAD2* and *TRIM28* genes.

Next, for each of the tested tumor types, we compared the mean mRNA‐SI score with the mean expression of BrD encoding genes. As presented in Figure 2C,D, we observed a robust positive association for 12 markers, namely *KAT2A*, *EP300*, *BRD1*, *SMARCA4*, *CECR2*, *ATAD2*, *BRD4*, *BRD2*, *BRDT*, *TRIM28*, *ZMYND8* and *BRWD3* genes, and a negative correlation for *KAT2B* and *SMARCA2*. These results strongly suggest that stemness‐like TCGA tumors (that exhibit high mean mRNA‐SI level) are significantly over‐expressing *KAT2A*, *SMARCA4*, *ATAD2* and *TRIM28*, and significantly underexpressing *KAT2B* and *SMARCA2*.

Previously, Malta et al^12^ have found a strong association between the mRNA‐SI and known clinical and molecular features of TCGA BRCA tumors, demonstrating that the basal subtype, known to exhibit an aggressive phenotype associated with an undifferentiated state, display the highest levels of mRNA‐SI. Therefore, we analyzed the expression of all BrD members in individual TCGA BRCA samples, stratified by molecular subtype (PAM50). As presented in Figure 2E, several BrD members, namely *BRPF1*, *SMARCA4*, *BRD7*, *BRD9*, *BAZ1B*, *ATAD2* and *BRD4* are significantly upregulated in basal breast cancer subtype (when compared to less aggressive luminal A and normal‐like subtype), which strongly mimics the results obtained for the mRNA‐SI (Table S5).

### 3.3 The expression of BrD family members in lower and higher‐grade tumors

**FIGURE 3:**
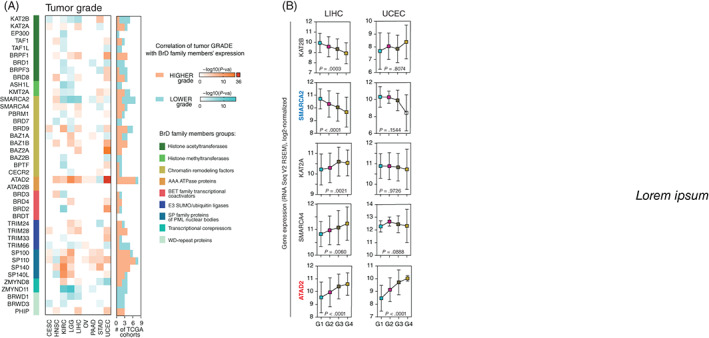
The expression of BrD family members in lower and higher grade tumors. (A) The association between BrD family members' expression and tumor grade—either lower (blue) or higher grade (orange), as determined with Spearman correlation test (−log10[*P*‐value]). (B) log2‐normalized expression of selected BrD family members in LIHC (left) and UCEC (right) tumors classified based on the “neoplasm histologic grade” feature from TCGA data into lower (G1, G2) and higher (G3, G4) grade tumors. The mean value with SD is plotted

As we have shown previously, higher‐grade tumors clearly exhibit stemness characteristics mirrored by elevated mRNA‐SI scores, especially in LIHC and UCEC (Figure S5).^38^ Therefore, we determined the association between the expression of BrD encoding genes and the tumor grade (Figure 3A). We observed significantly higher expression of *ATAD2* and significantly lower levels of *SMARCA2* in de‐differentiated tumors (Figure 3B), while the level of other previously selected BrD family members (*KAT2B*, *KAT2A* and *SMARCA4*) was relatively unchanged. Although we did not detect statistical significance in the analyses of IHC staining from the Human Protein Atlas^37^), we suggest that the level of ATAD2 protein is elevated while the level of SMARCA2 is depleted (Figure S6) in the higher grade LGG tumors, further supporting our first observation.

### 3.4 Stemness signature enrichment in the transcription profiles associated with the expression of BrD family members

**FIGURE 4:**
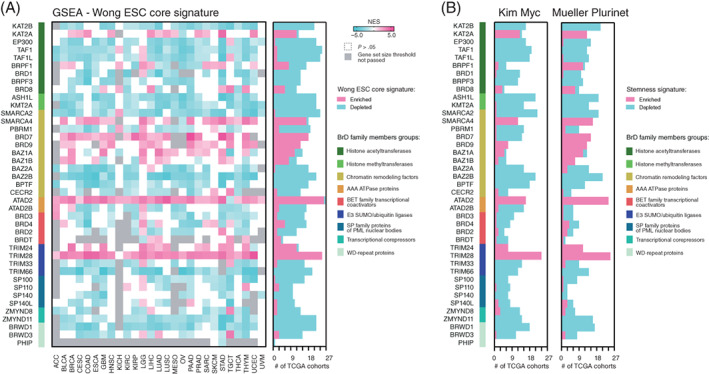
Stemness signature enrichment in the transcriptome profiles associated with the expression of BrD family members. (A) The gene set enrichment analysis (GSEA) using significantly DEGs (*P* < .05, FDR < 0.05) in TCGA patients divided into low‐expressing or high‐expressing BrD cohorts (using the mean expression of each BrD family member as a cut‐off) was performed with the stemness signature (Wong_ESC_Core) as a reference. The heatmap presents the normalized enrichment score (NES). White—no statistical significance (*P* > .05); gray—the gene set size thresholds were not reached or no DEGs were detected. (B) Similarly, the GSEA of BrD‐associated transcriptome profiles was performed with the stemness signature Kim Myc^39^ or Mueller Plurinet^40^ as a reference. The number of TCGA cohorts with either enriched (light pink) or depleted (light blue) stemness signatures are plotted

Next, using the mean expression of each BrD member as a cut‐off, we divided patients from all 27 solid tumor cohorts into BrD low‐expressing or high‐expressing groups. We further used all significantly DEGs between those groups to define the BrD‐related transcription profiles. The numbers of DEGs for each TCGA tumor type are presented in Figure S7. We used the GSEA to determine whether the BrD‐associated transcription profiles exhibit the enrichment of previously defined signatures of stemness.^10^, ^11^, ^12^, ^28^ For most BrD‐associated transcription profiles we observed significant depletion with stemness markers (Figure 4A) across distinct TCGA tumor types. As expected, we also detected a robust enrichment of *ATAD2*‐associated and *TRIM28*‐associated gene expression profiles with stemness signatures across most TCGA tumor types. Moreover, this was further validated with additional stem cell‐associated gene signatures (Figures 4B and S8).^39^, ^40^ As the association of *TRIM28* expression with cancer stemness was reported^22^, ^25^ and the versatility of this phenomenon was demonstrated recently,^38^ we have further focused on other BrD family members.

To unequivocally confirm the enrichment of selected BrD‐associated transcriptome profiles with the stemness signatures, we used additional GEO datasets in our studies (Table S4). The results presented in Figure S9 further validated our first observation of *ATAD2*‐associated transcriptome profiles being significantly enriched with stemness signatures regardless of the tumor type.

### 3.5 Targets for E2F and c‐Myc transcription factors are significantly enriched in ATAD2, SMARCA4 and KAT2A associated expression profiles

**FIGURE 5:**
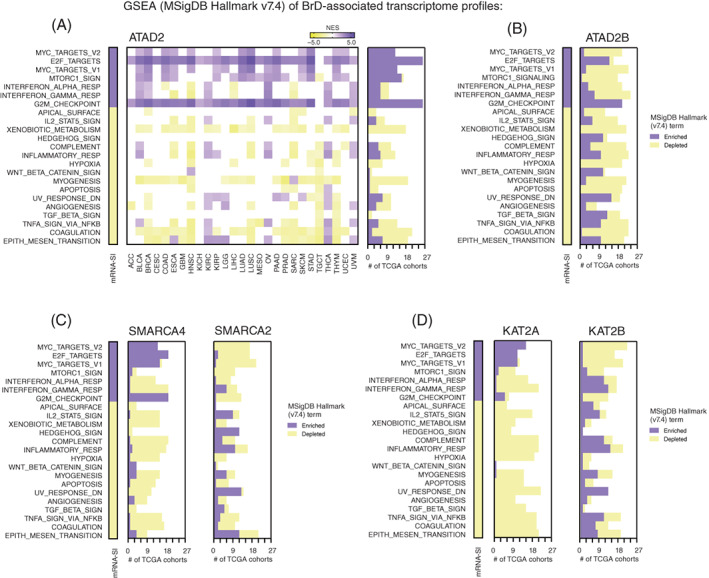
The transcriptome profiles associated with the expression of selected BrD family members are enriched with c‐Myc and E2F transcription factor targets that significantly mirror the enrichment of mRNA‐SI gene signature with the “Hallmarks of cancer” terms. (A) The GSEA using significantly DEGs (*P* < .05, FDR < 0.05) in TCGA patients divided into low‐expressing or high‐expressing *ATAD2* cohorts (using the mean expression of *ATAD2* as a cut‐off) was performed with the MSigDB Hallmark (v7.4) collection as a reference. The heatmap presents the normalized enrichment score (NES). White—no statistical significance (*P* > .05) or no DEGs were detected. Only those Hallmark terms, that were previously determined as significantly enriched (violet) or depleted (yellow) in the mRNA‐SI gene signature are presented in the heatmap. Right panel denotes the number of TCGA cohorts with either enriched (violet) or depleted (yellow) Hallmark term. (B‐D) Similarly, the GSEA of *ATAD2B* (B), *SMARCA4* (C, left) and *SMARCA2* (C, right), *KAT2A* (D, left), *KAT2B* (D, right) was performed with the MSigDB Hallmark (v7.4) collection as a reference. The number of TCGA cohorts with either enriched (violet) or depleted (yellow) MSigDB Hallmark terms are plotted

Recently, Malta et al^12^ have demonstrated that stemness‐associated expression profiles are significantly enriched with targets for c‐Myc (MYC proto‐oncogene) transcription factor, and depleted with markers of hypoxia, Wnt/β‐catenin signaling, tumor growth factor‐β (TGF‐β) signaling and epithelial‐mesenchymal transition (EMT). Here, we performed the GSEA analysis of mRNA‐SI gene signature (Figure S10) to define the top enriched or depleted “hallmarks of cancer” terms (FDR < 0.05) and observed a significant enrichment of c‐Myc and E2F transcription factor target genes in the mRNA‐SI gene signature. Subsequently, using the GSEA tool and the MSigDB Hallmark (v7.4) dataset as a reference, we observed that *ATAD2*‐associated transcription profiles are also significantly enriched with the targets for E2F and c‐Myc transcription factors. Moreover, we detected a significant enhancement of cell cycle‐related term “G2/M checkpoint” in ATAD2^HIGH^ expressing tumors across all tested TCGA tumor types (Figure 5A). To confirm that this is specific for *ATAD2*, we also analyzed the *ATAD2B*‐related expression profiles and observed that these are barely enriched with the targets for c‐Myc transcription factors, although still exhibiting significant enrichment of E2F targets and G2/M checkpoint‐related genes (Figures 5B and S11A).

Similarly, we observed opposite results for *SMARCA2* and *SMARCA4*‐related gene expression profiles (Figures 5C and S11B,C) as well as for *KAT2A* and *KAT2B*‐associated transcription profiles (Figures 5D and S11D,E), with *SMARCA4*‐related and *KAT2A*‐related gene expression profiles exhibiting significant enrichment of c‐Myc and E2F transcription factors' target genes.

Next, we looked at the association between selected BrD members and c‐Myc and E2F family transcription factors and observed that only ATAD2 (according to the pathway commons protein‐protein interactions dataset) interacts with c‐Myc transcription factor and that the expression of *ATAD2* positively correlated with the *MYC* level across TCGA tumor types (Figure S12A‐D), while for other BrD members, the association was rather tumor‐specific. Also, the TCGA tumors with high average *ATAD2* levels express significantly higher levels of *E2F1* and *E2F2* transcription factors (Figure S12E‐G), which together might explain the enrichment of *ATAD2*‐associated gene expression profiles with the targets for c‐Myc and E2F transcription factors. *ATAD2* and *MYC* are both encoded within the long arm of chromosome 8, therefore we excluded that the abovementioned association results strictly from shared localization. As presented in Figure S13, results obtained for *FBXO32*, *ZHX1* and *ANXA13* genes encoded within the same region as *ATAD2* clearly emphasize the specificity of *ATAD2* and *MYC* as well as *ATAD2* and cancer stemness associations.

All the abovementioned results strongly support our claim that *ATAD2* is positively associated with cancer stemness, regardless of the tumor type and this association might be mediated at least partially by the interaction with the c‐Myc transcription factor—an essential factor facilitating the acquisition and maintenance of stem cell properties.

## 4 DISCUSSION

This is the first report of the association between distinct BrD proteins and cancer de‐differentiation status across different types of solid tumors. Here, we used transcriptomic data from TCGA and GEO and harnessed several publicly available bioinformatic platforms or tools to demonstrate that: (a) most BrD members exhibit differential expression in tumor and normal tissues and the expression pattern is protein‐specific and highly depends on the tumor type; (b) the association between BrD proteins expression and cancer stemness is mostly negative, with only several proteins being consistently positively correlated with cancer de‐differentiation status regardless of the tumor type; (c) higher‐grade tumors of different types express significantly higher levels of *ATAD2* and lower levels of *SMARCA2*; (d) the transcriptome profiles associated with high expression of *ATAD2*, *SMARCA4* or *KAT2A* are significantly enriched with stemness signatures; (e) *ATAD2*‐associated gene expression profiles display significant enrichment with the c‐Myc targets that mirrors the enrichment observed for the mRNA‐SI gene signature; (f) *ATAD2* and *MYC* are commonly upregulated in cancer tissues and together might regulate the acquisition of stem cell‐like phenotype by solid tumors.

This is the first report that comprehensively analyzes the association of BrD proteins with cancer de‐differentiation status across numerous types of solid tumors based on previously reported stemness quantifiers: the mRNA‐SI—the transcriptome‐based stemness index developed by the machine learning algorithm^12^ using a significant number of molecular profiles of distinct stem cell populations and their differentiated progeny, as well as other stem cell gene signatures, derived from transcriptional profiling of undifferentiated normal stem cells.^10^, ^11^, ^28^, ^39^, ^40^ The transcriptional program previously recognized in normal stem cells is commonly launched by different human epithelial cancers, which strongly suggests its' prevalence in gaining cancer stemness regardless of the tumor type, and stemness signatures were demonstrated as very efficient in quantifying cancer stemness (commonly reflected in the histopathological grade).^10^, ^28^

To date, only several members of BrD family were directly connected with cancer stemness. Especially, the engagement of BRD4, a member of BET transcriptional coactivators, as well as the role for TRIM28—a transcriptional co‐repressor, known to mediate E3 SUMO/ubiquitin ligase activity, have been well established in mediating the self‐renewal properties of cancer stem cells.^15^, ^16^, ^17^, ^18^, ^19^, ^20^, ^21^, ^22^, ^23^, ^24^, ^25^, ^26^, ^27^

We have recently reported that *TRIM28* overexpression closely associates with cancer stemness in breast cancer and melanomas and subsequently demonstrated that this phenomenon is very universal across diverse types of solid tumors.^22^, ^25^, ^38^ Several potential modes of actions for TRIM28 in obtaining cancer stemness have been suggested including (a) transcriptional co‐repression of differentiating genes^27^ followed by (b) the enhancement of stem cell markers' expression.^41^ Also, TRIM28 might act (c) by targeting for proteasomal degradation (through RING‐mediated E3 ubiquitin ligase activity) various proteins, that is, AMPK, a “metabolic switch” that attenuates cancer stemness.^22^ As for BRD4, Venkataraman et al^17^ have demonstrated an indisputable role in mediating the self‐renewal of cancer cells in c‐Myc‐driven medulloblastomas, which was further observed in gliomas,^16^ stomach,^20^ and liver tumors.^19^ Similar to TRIM28, the exact mechanism of BRD4‐associated cancer stemness‐high phenotype is not clear, although unequivocally it depends on bromodomain activity. Surprisingly, using the TCGA transcriptomic data we did not observe a significant association of *BRD4* expression with cancer stemness across 27 tested tumor types, in contrast to previously reported *TRIM28* and newly discovered *ATAD2* (and to some part also *SMARCA4*) and cancer de‐differentiation status.

*ATAD2*, a chromatin modulator that possesses an AAA+ ATPase domain and a bromodomain, is normally overexpressed in nonspecialized cells, including embryonic stem cells, and in germ cells. Recently, ATAD2 has been recognized as essential in supporting specific transcriptional programs in ESC cells, modulating their proliferation and differentiation.^42^ Here, using the TCGA and GEO transcriptomic data, we report yet unrecognized association between *ATAD2* overexpression and cancer stemness in solid tumors across distinct tumor types.

Several studies have previously reported significant upregulation of *ATAD2* expression in solid tumors of distinct origins as well as its association with poor patients' outcome, especially in lung, breast, liver, ovarian and cervix cancers.^43^ Our results demonstrate that a high *ATAD2* level is significantly associated with a worse outcome in ACC, KIRP, LGG, LUAD, MESO, PAAD TCGA tumors, strongly suggesting that *ATAD2* overexpression favors malignant transformation of unrelated cancer types. As previously reported, a high *ATAD2* expression correlated with more aggressive tumor subgroups of cervical,^43^ colorectal,^44^ gastric^45^ and liver cancer patients,^46^ although a direct link with a cancer stem cell compartment was not tested. Here, we demonstrate that *ATAD2* upregulation positively correlates with a higher tumor grade of HNSC, KIRC, LGG, LIHC, OV, PAAD and UCEC tumors, and higher‐grade tumors clearly display stem cell features, particularly stemness‐related gene expression profiles.

Moreover, a significant correlation between the mean *ATAD2* expression and the mean mRNA‐SI score across tested tumor types suggests that strongly de‐differentiated tumors overexpress *ATAD2*. We report yet unrecognized correlation between *ATAD2* upregulation and significant enrichment of stem cell‐like phenotype in cancer and prove its' versatility across solid tumors. The transcriptome profiles of *ATAD2* ^HIGH^ cancers are robustly overrepresented with predefined stemness gene signatures as well as with the targets for E2F and c‐Myc transcription factors. Previously, ATAD2 has been identified as a transcriptional co‐regulator modulating the expression of estrogen and androgen receptors or E2F and c‐Myc transcription factors, all known as cancer/proliferation‐promoting factors.^47^ The pRB‐E2F pathway tightly regulates *ATAD2* expression, which is essential for the growth of normal and cancer cells. As a direct binding partner for both E2F and c‐Myc, ATAD2 induces the expression of genes that facilitate cell cycle progression and inhibition of apoptosis in many different types of cancers, including breast, lung and prostate tumors.^48^ Also, Wu et al^49^ proposed that ATAD2 might cooperate with the c‐Myc to control the level of *SMO* and *GLI1*, leading to the Hedgehog (Hh) pathway and feedback of the Hh pathway activation in liver tumor cells. Similar results were recently reported for esophagus tumors. Li et al^50^ suggested that ATAD2 could regulate cancer stem cell biological features by activating the Hh pathway, as silencing of *ATAD2* decreased the proliferation, invasion, migration and colony formation abilities of CSCs which corresponds to the Hh pathway inhibition.

Our results are in line with previously reported *ATAD2* and *MYC* co‐expression. c‐Myc dysregulation accounts for most of the similarities between aggressive tumors and normal stem cell characteristics. Therefore, we suggest that the ATAD2‐related cancer stem cell‐like phenotype is mediated through both ATAD2 and c‐Myc proteins and propose ATAD2 as a druggable target for de‐differentiated tumors (especially those overexpressing *MYC*), which emerges achievable when considering its ATPase activity and its bromodomain. However, molecular studies are indispensable in order to determine the exact role for ATAD2 in cancer stem cell‐like phenotype of solid tumors.

## 5 CONCLUSIONS

To conclude, our results demonstrate that BrD family genes display diverse expression patterns in stem cell‐like solid tumors. Among all tested BrD proteins, the newly discovered positive association between *ATAD2* and cancer de‐differentiation status emerges as universal regardless of the tumor type. Higher‐grade tumors display significant upregulation of *ATAD2* expression and high *ATAD2* level corresponds to enhanced c‐Myc transcriptional activity. Together, we suggest that ATAD2 might serve as a potential therapeutic target for de‐differentiated solid tumors that strongly exhibit cancer stem cell‐like characteristics.

## CONFLICT OF INTEREST

The authors declare no conflict of interests.

## AUTHOR CONTRIBUTIONS

Patrycja Czerwinska designed the experiments and analyses. All the authors participated in data acquisition. Patrycja Czerwinska, Anna Maria Jaworska and Nikola Agata Wlodarczyk performed data analyses, interpretation and validation. Patrycja Czerwinska visualized all the results and drafted the manuscript. All the authors reviewed the manuscript and approved its final version. Patrycja Czerwinska and Andrzej Adam Mackiewicz supervised the work.

## ETHICS STATEMENT

The study is based on publicly available datasets and does not need any ethics committee's agreement. The study does not violate the rights of other persons or institutions.

## Supporting information


**Appendix S1** Supporting Information.

## Data Availability

Only publicly available data were used in this study, and data sources and handling of these data are described in the Materials and Methods and in the Supporting Information Material. Further information is available from the corresponding author upon request.
