# Supplementary material for: The association between bromodomain proteins and cancer stemness in different solid tumor types
Source: Int J Cancer. 2022 Jan 29;150(11):1838–49. doi: 10.1002/ijc.33937 (PMC9303422; doi:10.1002/ijc.33937)
Supplement: Supplementary file 1 — Appendix S1 Supporting Information. [file IJC-150-1838-s001.pdf]

Supplementary File

The association between bromodomain (BrD) proteins and cancer stemness in different solid tumor types

Czerwinska Patrycja, Jaworska Anna Maria, Wlodarczyk Nikola Agata, Cisek Malgorzata, Karwacka Marianna, Lipowicz Julia, Ostapowicz Julia, Rosochowicz Monika, Mackiewicz Andrzej Adam

Table of content:

Supplementary Materials and Methods 2
Supplementary Table 1 3
Supplementary Table 2 4
Supplementary Table 3 7
Supplementary Table 4 39
Supplementary Table 5 40
Supplementary Figure 1. 43
Supplementary Figure 2. 44
Supplementary Figure 3. 45
Supplementary Figure 4. 47
Supplementary Figure 5. 48
Supplementary Figure 6. 49
Supplementary Figure 7. 50
Supplementary Figure 8. 51
Supplementary Figure 9. 52
Supplementary Figure 10. 53
Supplementary Figure 11. 54
Supplementary Figure 12. 55
Supplementary Figure 13. 56
Supplementary References 58

Supplementary Materials and Methods

The expression of BrD family members in the Oncomine datasets

Data regarding BrD family members' expression in various cancer types was retrieved from the online database, Oncomine (https://www.oncomine.org/resource/login.html) (1). This platform contains a large collection of independent datasets and expertly curated data. It can be used to identify novel targets for drug development and to interrogate gene expression profiles along with the clear and consistent interpretation of results. Differences in mRNA expression between cancer tissues and their normal tissue counterparts were calculated using the following threshold parameters: p-value < 0.01, fold-change > 2, and gene ranking in the top 10%.

Prognosis analysis using the Prognoscan database

The PrognoScan (http://dna00.bio.kyutech.ac.jp/PrognoScan/) (2) database was used for the meta-analysis of the prognostic value of various genes. This online platform assists in investigating the relationship between gene expression and patient prognosis across a large collection of cancer microarray datasets. The correlation between BrD family members' expression and survival was investigated in several cancer types using this tool. The significance threshold was adjusted to a Cox p-value < 0.05. These results are briefly presented in Supplementary Table 3 (p. 7-38).

The Human Protein Atlas

The representative results of immunohistochemistry staining of LGG samples with anti-ATAD2 (HPA043495) and anti-SMARCA2 (HPA064033) antibodies were downloaded from the Human Protein Atlas database (www.proteinatlas.org) (3). Samples presented in this study: (i) for ATAD2 IHC - Patient ID (PID) 3023, 2907; (ii) for SMARCA2 IHC - PID 2914, 2910.

Supplementary Table 1
TCGA cohorts included in this study.

Abbreviation	TCGA tumor type	Number of samples with RNA-Seq (V2 RSEM), mRNA-SI score, and survival data
ACC	Adrenocortical Carcinoma	78
BLCA	Bladder Urothelial Carcinoma	402
BRCA	Breast Invasive Carcinoma	1083
CESC	Cervical Squamous Cell Carcinoma and Endocervical Adenocarcinoma	301
COAD	Colorectal Adenocarcinoma	275
ESCA	Esophageal Carcinoma	182
GBM	Glioblastoma Multiforme	148
HNSC	Head and Neck Squamous Cell Carcinoma	514
KICH	Kidney Chromophobe	65
KIRC	Kidney Renal Clear Cell Carcinoma	515
KIRP	Kidney Renal Papillary Cell Carcinoma	266
LGG	Brain Lower Grade Glioma	512
LIHC	Liver Hepatocellular Carcinoma	367
LUAD	Lung Adenocarcinoma	499
LUSC	Lung Squamous Cell Carcinoma	480
MESO	Mesothelioma	87
OV	Ovarian Serous Cystadenocarcinoma	269
PAAD	Pancreatic Adenocarcinoma	156
PRAD	Prostate Adenocarcinoma	493
SARC	Sarcoma	252
SKCM	Skin Cutaneous Melanoma	468
STAD	Stomach Adenocarcinoma	392
TGCT	Testicular Germ Cell Tumor	133
THCA	Thyroid Carcinoma	499
THYM	Thymoma	499
UCEC	Uterine Corpus Endometrial Carcinoma	173
UVM	Uveal Melanoma	68

Supplementary Table 2
Bromodomain family members.

Group	Name	Synonyms	Name	# of BrDs
**group I - HAT (histone acetyltransferases)**	**KAT2B**	CAF, PCAF, GCN5L	P300/CBP-associated factor	1
	**KAT2A**	GCN5, GCN5L2, HGCN5, PCAF-b	General control of amino acid synthesis protein 5-like 2	1
	**EP300**	P300, KAT3B, RSTS2, p300, MKHK2	E1A-associated protein p300	1
	**TAF1**	BA2R, CCG1, CCGS, TAF2A, TAF(ii)250, DYT3, DYT3/KAT4, N-NSCL2, OF, P250, TAF2A, TAFII-250, TAFII250, XDP, MRXS33	Transcription initiation factor TFIID subunit 1	2
	**TAF1L**	TAF(ii)210, TAF2A2	Transcription initiation factor TFIID subunit 1-like	2
	**BRPF1**	BR140, Peregrin, IDDDFP	Bromodomain and PHD finger- containing protein 1	1
	**BRD1**	BRL, BRPF2	Bromodomain-containing protein 1	1
	**BRPF3**	KIAA1286	Bromodomain and PHD finger- containing protein 3	1
	**BRD8**	SMAP, SMAP2, p120, TrCP120	Bromodomain-containing protein 8	2
**group II - histone methyltransferases**	**ASH1L**	ASH1, KMT2H, KIAA1420	Absent small and homeotic disks protein 1 homolog	1
	**MLL**	KMT2A, ALL1, CXXC7, HRX, HTRX, MLL1, TRX1, ALL-1, MLL1A, TRX1, WDSTS	Myeloid/lymphoid or mixed-lineage leukaemia	1
**group III - chromatin remodeling factors**	**SMARCA2**	BAF190, BAF190B, BRM, NCBRS, SNF2, SNF2L2, SNF2LA, SWI2, Sth1p, hBRM, hSNF2a, NCBRS, SND2A	SWI/SNF-related matrix-associated actin-dependent regulator of chromatin subfamily A member 2	1

	**SMARCA4**	BRG1, CSS4, SNF2, SWI2, MRD16, RTPS2, BAF190, SNF2L4, SNF2LB, hSNF2b, BAF190A, SNF2-beta	SWI/SNF-related matrix-associated actin-dependent regulator of chromatin subfamily A member 4	1
	**PBRM1**	PB1, BAF180, HPB1	Polybromo-1	6
	**BRD7**	BP75, CELTIX1, NAG4	Bromodomain-containing protein 7	1
	**BRD9**	LAVS3040, FLJ13441, PRO985	Bromodomain-containing protein 9	1
	**BAZ1A**	ACF1, WCRF180, hWALp1, WALp1	Bromodomain adjacent to zinc finger domain protein 1A	1
	**BAZ1B**	WBSC10, WBSCR10, WBSCR9, WSTF, HWALp2	Bromodomain adjacent to zinc finger domain protein 1B	1
	**BAZ2A**	KIAA0314, TIP5, WALp3, HWALp3	Bromodomain adjacent to zinc finger domain protein 2A	1
	**BAZ2B**	hWALp4, KIAA1476, WALp4	Bromodomain adjacent to zinc finger domain protein 2B	1
	**BPTF**	FAC1, FALZ, NURF301, NEDDFL	Bromodomain and PHD finger- containing transcription factor	1
	**CECR2**	KIAA1740	Cat eye syndrome critical region protein 2	1
**group IV - AAA ATPase proteins**	**ATAD2**	ANCCA, CT137, PRO2000	ATPase family AAA domain-containing protein 2	1
	**ATAD2B**	KIAA1240	ATPase family AAA domain-containing protein 2B	1
**group V - BET family transcriptional coactivators**	**BRD3**	KIAA0043 RING3L, ORFX	Bromodomain-containing protein 3	2
	**BRD4**	HUNK1, CAP, HUNKI, MCAP	Bromodomain-containing protein 4	2
	**BRD2**	KIAA9001, RING3, BRD2-IT1, D6S113E, FSH, FSRG1, NAT, RNF3	Bromodomain-containing protein 2	2
	**BRDT**	BRD6, CT9, SPGF21	Bromodomain testis-specific protein	2
**group VI - E3 SUMO/ubiquitin ligases**	**TRIM24**	RNF82, TIF1, TIF1α, TIF1A, PTC6	Transcription intermediary factor 1-alpha	1

	**TRIM28**	KAP1, RNF96, TIF1β, PPP1R157, TIF1B, KRIP1	Transcription intermediary factor 1-beta	1
	**TRIM33**	KIAA1113, RFG7, TIF1γ, ECTO, PTC7, TIF1G	Transcription intermediary factor 1-gamma	1
	**TRIM66**	TIF1D, TIF1δ, C11orf29, KIAA0298	Tripartite motif-containing protein 66	1
**group VII - SP family proteins of PML nuclear bodies**	**SP100**	Lysp100b	Nuclear autoantigen Sp-100	1
	**SP110**	IFI41, IFI75, IPR1, VODI	Sp110 nuclear body protein	1
	**SP140**	LYSP100, LYSP100-A, LYSP100-B	Nuclear body protein SP140	1
	**SP140L**	LOC93349	Nuclear body protein SP140-like protein	1
**group VIII - transcriptional corepressors**	**ZMYND8**	KIAA1125, PRKCBP1, RACK7, PRO2893	Zinc finger MYND domain-containing protein 8, protein kinase C-binding protein 1	1
	**ZMYND11**	BS69, BRAM1, MDR30	Zinc finger MYND domain-containing protein 11	1
**group IX - WD-repeat proteins**	**BRWD1**	C21orf107, DCAF19, N143, WDR9, WRD9, FLJ11315	Bromodomain and WD repeat-containing protein 1	2
	**BRWD3**	BRODL, MRX93, FLJ38568	Bromodomain and WD repeat-containing protein 3	2
	**PHIP**	WDR11, BRWD2, CHUJANS, DCAF14, DIDOD, WDR11, Ndrp	PH-interacting protein	2

Supplementary Table 3
Datasets from the Prognoscan database.

ASH1L												
**Lp.**	**DATASET**	**CANCER TYPE**	**SUBTYPE**	**COHORT**	**AUTHOR**	**ARRAY TYPE**	**PROBE ID**	**N**	**CUTPOINT**	**COX P-VALUE**	**ln(HR)**	**HR [95% CI low - upp]**
**Worse prognosis**												
1	GSE11595	Esophagus cancer	Adenocarcinoma	Sutton	Giddings	CRUKDMF_22K_v1,0,0	229884	34	0.59	0.049276	**1.55**	4,71 [1,00 - 22,10]
**Better prognosis**												
2	GSE13507	Bladder cancer		CNUH	Kim	Human-6 v2	ILMN_1782032	165	0.32	0.019384	**-1.7**	0,18 [0,04 - 0,76]
3	GSE31210	Lung cancer	Adenocarcinoma	NCCRI	Okayama	HG-U133_Plus_2	222667_s_at	204	0.67	0.002221	**-2.31**	0,10 [0,02 - 0,44]
4	GSE31210	Lung cancer	Adenocarcinoma	NCCRI	Okayama	HG-U133_Plus_2	226447_at	204	0.2	0.002087	**-2.4**	0,09 [0,02 - 0,42]
**ATAD2**												
**Lp.**	**DATASET**	**CANCER TYPE**	**SUBTYPE**	**COHORT**	**AUTHOR**	**ARRAY TYPE**	**PROBE ID**	**N**	**CUTPOINT**	**COX P-VALUE**	**ln(HR)**	**HR [95% CI low - upp]**
**Worse prognosis**												
1	GSE12945	Colorectal cancer		Berlin	Staub	HG-U133A	218782_s_at	62	0.85	0.002771	**3.14**	23,00 [2,95 - 179,31]
2	GSE19234	Skin cancer	Melanoma	NYU	Bogunovic	HG-U133_Plus_2	218782_s_at	38	0.89	0.000047	**2.28**	9,74 [3,25 - 29,12]
3	GSE19234	Skin cancer	Melanoma	NYU	Bogunovic	HG-U133_Plus_2	235266_at	38	0.53	0.003717	**1.65**	5,20 [1,71 - 15,86]
4	GSE19234	Skin cancer	Melanoma	NYU	Bogunovic	HG-U133_Plus_2	222740_at	38	0.82	0.000703	**1.64**	5,14 [1,99 - 13,24]
5	GSE19234	Skin cancer	Melanoma	NYU	Bogunovic	HG-U133_Plus_2	228401_at	38	0.53	0.006564	**1.13**	3,10 [1,37 - 7,00]
6	GSE4271-GPL97	Brain cancer	Astrocytoma	MDA	Phillips	HG-U133B	222740_at	77	0.75	0.000191	**0.92**	2,51 [1,55 - 4,07]
7	GSE4271-GPL97	Brain cancer	Astrocytoma	MDA	Phillips	HG-U133B	228401_at	77	0.58	0.000261	**0.87**	2,39 [1,50 - 3,82]
8	jacob-00182-CANDF	Lung cancer	Adenocarcinoma	CAN/DF	Shedden	HG-U133A	218782_s_at	82	0.89	0.024631	**0.67**	1,96 [1,09 - 3,53]

9	GSE4271-GPL96	Brain cancer	Astrocytoma	MDA	Phillips	HG-U133A	218782_s_at	77	0.61	0.002957	**0.63**		1,88 [1,24 - 2,84]
10	GSE1456-GPL96	Breast cancer		Stockholm (1994-1996)	Pawitan	HG-U133A	218782_s_at	159	0.23	0.00619	**0.61**		1,84 [1,19 - 2,84]
11	GSE31210	Lung cancer	Adenocarcinoma	NCCRI	Okayama	HG-U133_Plus_2	218782_s_at	204	0.53	0.0413	**0.57**		1,77 [1,02 - 3,06]
12	GSE1456-GPL97	Breast cancer		Stockholm (1994-1996)	Pawitan	HG-U133B	228401_at	159	0.59	0.024559	**0.53**		1,70 [1,07 - 2,71]
13	GSE4271-GPL97	Brain cancer	Astrocytoma	MDA	Phillips	HG-U133B	235266_at	77	0.42	0.015565	**0.52**		1,67 [1,10 - 2,54]
14	GSE1456-GPL97	Breast cancer		Stockholm (1994-1996)	Pawitan	HG-U133B	222740_at	159	0.33	0.01076	**0.52**		1,69 [1,13 - 2,52]
15	GSE13213	Lung cancer	Adenocarcinoma	Nagoya (1995-1999, 2002-2004)	Tomida	G4112F	A_23_P216068	117	0.87	0.000073	**0.52**		1,69 [1,30 - 2,19]
16	GSE13507	Bladder cancer		CNUH	Kim	Human-6 v2	ILMN_1763064	165	0.36	0.002315	**0.39**		1,47 [1,15 - 1,88]
17	GSE13213	Lung cancer	Adenocarcinoma	Nagoya (1995-1999, 2002-2004)	Tomida	G4112F	A_24_P59596	117	0.87	0.021434	**0.38**		1,46 [1,06 - 2,00]
18	GSE17260	Ovarian cancer		Niigata (1997-2008)	Yoshihara	G4112A	A_23_P216068	110	0.45	0.049273	**0.32**		1,37 [1,00 - 1,89]
19	GSE9891	Ovarian cancer		AOCS, RBH, WH, NKI-AVL (1992-2006)	Tothill	HG-U133_Plus_2	228401_at	278	0.33	0.010738	**0.29**		1,34 [1,07 - 1,67]
20	GSE9891	Ovarian cancer		AOCS, RBH, WH, NKI-AVL (1992-2006)	Tothill	HG-U133_Plus_2	222740_at	278	0.26	0.037231	**0.19**		1,21 [1,01 - 1,44]
**ATAD2B**													

Lp.	DATASET	CANCER TYPE	SUBTYPE	COHORT	AUTHOR	ARRAY TYPE	PROBE ID	N	CUTP OINT	COX P-VALUE	ln(HR)	HR [95% CI low - upp]
**Worse prognosis**												
1	GSE17537	Colorectal cancer		VMC	Smith	HG-U133_Plus_2	213387_at	55	0.6727 27	0.012318 2	**1.09674**	2,99 [1,27 - 7,07]
**Better prognosis**												
2	DUKE-OC	Ovarian cancer		Duke	Bild	HG-U133A	213387_at	133	0.8646 62	0.030379 3	**-0.343314**	0,71 [0,52 - 0,97]
3	GSE9891	Ovarian cancer		AOCS, RBH, WH, NKI-AVL (1992-2006)	Tothill	HG-U133_Plus_2	1558807_at	278	0.8201 44	0.038556 3	**-0.928688**	0,40 [0,16 - 0,95]
**BAZ1A**												
**Lp.**	**DATASET**	**CANCER TYPE**	**SUBTYPE**	**COHORT**	**AUTHOR**	**ARRAY TYPE**	**PROBE ID**	**N**	**CUTP OINT**	**COX P-VALUE**	**ln(HR)**	**HR [95% CI low - upp]**
**Worse prognosis**												
1	GSE13213	Lung cancer	Adenocarcinoma	Nagoya (1995-1999, 2002-2004)	Tomida	G4112F	A_23_P7679 9	117	0.8803 42	0.001190 77	**0.514555**	1,67 [1,23 - 2,28]
**Better prognosis**												
2	DUKE-OC	Ovarian cancer		Duke	Bild	HG-U133A	217985_s_at	133	0.1203 01	0.037874 5	**-0.253009**	0,78 [0,61 - 0,99]
3	DUKE-OC	Ovarian cancer		Duke	Bild	HG-U133A	217986_s_at	133	0.1879 7	0.000424 7	**-0.438991**	0,64 [0,51 - 0,82]
4	jacob-00182-CANDF	Lung cancer	Adenocarcinoma	CAN/DF	Shedden	HG-U133A	217985_s_at	82	0.4024 39	0.039795 1	**-0.659467**	0,52 [0,28 - 0,97]
5	GSE16131 -GPL96	Blood cancer	Follicular lymphoma	NCI (1974-2001)	Dave	HG-U133A	217986_s_at	180	0.4388 89	0.011553 4	**-0.736501**	0,48 [0,27 - 0,85]
**BAZ1B**												
**Lp.**	**DATASET**	**CANCER TYPE**	**SUBTYPE**	**COHORT**	**AUTHOR**	**ARRAY TYPE**	**PROBE ID**	**N**	**CUTP OINT**	**COX P-VALUE**	**ln(HR)**	**HR [95% CI low - upp]**
**Worse prognosis**												

1	GSE16581	Brain cancer	Meningioma	UCLA	Lee	HG-U133_Plus_2	211313_s_at	67	0.447761	0.034414	**3.85135**	47,06 [1,33 - 1669,26]
2	GSE4475	Blood cancer	B-cell lymphoma	Berlin (2003-2005)	Hummel	HG-U133A	211313_s_at	158	0.626582	0.00489778	**1.40684**	4,08 [1,53 - 10,88]
3	GSE19234	Skin cancer	Melanoma	NYU	Bogunovic	HG-U133_Plus_2	213336_at	38	0.868421	0.0395952	**1.25822**	3,52 [1,06 - 11,66]
4	GSE8841	Ovarian cancer		Milan (1992-2003)	Marchini	G4100A	16699	81	0.419753	0.0152455	**1.10992**	3,03 [1,24 - 7,44]
5	GSE4412-GPL97	Brain cancer	Glioma	UCLA (1996-2003)	Freije	HG-U133B	229658_at	74	0.851351	0.0312112	**0.916118**	2,50 [1,09 - 5,75]
6	MGH-glioma	Brain cancer	Glioma	CBTTB, MGH, BWH, CH	Nutt	HG-U95A	32261_at	50	0.58	0.00305019	**0.482752**	1,62 [1,18 - 2,23]
7	DUKE-OC	Ovarian cancer		Duke	Bild	HG-U133A	213336_at	133	0.894737	0.0191595	**0.454304**	1,58 [1,08 - 2,30]
8	DUKE-OC	Ovarian cancer		Duke	Bild	HG-U133A	211313_s_at	133	0.609023	0.00993188	**0.439666**	1,55 [1,11 - 2,17]
**Better prognosis**												
9	jacob-00182-CANDF	Lung cancer	Adenocarcinoma	CAN/DF	Shedden	HG-U133A	213336_at	82	0.792683	0.0359286	**-0.676727**	0,51 [0,27 - 0,96]
10	GSE31210	Lung cancer	Adenocarcinoma	NCCRI	Okayama	HG-U133_Plus_2	213336_at	204	0.196078	0.00442138	**-0.806937**	0,45 [0,26 - 0,78]
11	GSE31210	Lung cancer	Adenocarcinoma	NCCRI	Okayama	HG-U133_Plus_2	229658_at	204	0.735294	0.0159109	**-0.915515**	0,40 [0,19 - 0,84]
12	GSE1456-GPL97	Breast cancer		Stockholm (1994-1996)	Pawitan	HG-U133B	229658_at	159	0.45283	0.0310513	**-0.986347**	0,37 [0,15 - 0,91]
13	GSE17537	Colorectal cancer		VMC	Smith	HG-U133_Plus_2	213336_at	55	0.218182	0.0300931	**-1.66332**	0,19 [0,04 - 0,85]
**BAZ2A**												
**Lp.**	**DATASET**	**CANCER TYPE**	**SUBTYPE**	**COHORT**	**AUTHOR**	**ARRAY TYPE**	**PROBE ID**	**N**	**CUTPOINT**	**COX P-VALUE**	**ln(HR)**	**HR [95% CI low - upp]**
**Worse prognosis**												
1	DUKE-OC	Ovarian cancer		Duke	Bild	HG-U133A	215437_x_at	133	0.481203	0.0209704	**2.22373**	9,24 [1,40 - 61,05]

2	GSE4475	Blood cancer	B-cell lymphoma	Berlin (2003-2005)	Hummel	HG-U133A	201355_s_at	158	0.601266	0.0427007	**1.72127**	5,59 [1,06 - 29,55]
3	DUKE-OC	Ovarian cancer		Duke	Bild	HG-U133A	201355_s_at	133	0.360902	0.00117896	**1.11142**	3,04 [1,55 - 5,95]
4	GSE14814	Lung cancer	NSCLC	JRB,10	Zhu	HG-U133A	201353_s_at	90	0.744444	0.0455862	**0.963538**	2,62 [1,02 - 6,74]
5	GSE4412-GPL96	Brain cancer	Glioma	UCLA (1996-2003)	Freije	HG-U133A	201355_s_at	74	0.189189	0.036324	**0.802319**	2,23 [1,05 - 4,73]
6	GSE3141	Lung cancer	NSCLC	Duke	Bild	HG-U133_Plus_2	201355_s_at	111	0.558559	0.0339139	**0.612451**	1,84 [1,05 - 3,25]
7	E-TABM-346	Blood cancer	DLBCL	GELA (1998-2000)	Jais	HG-U133A	215437_x_at	53	0.716981	0.00939897	**0.606825**	1,83 [1,16 - 2,90]
8	GSE16131-GPL96	Blood cancer	Follicular lymphoma	NCI (1974-2001)	Dave	HG-U133A	201355_s_at	180	0.316667	0.032089	**0.575004**	1,78 [1,05 - 3,01]
9	GSE5122	Blood cancer	AML	San Diego	Raponi	HG-U133A	215437_x_at	58	0.310345	0.0493598	**0.353637**	1,42 [1,00 - 2,03]
**Better prognosis**												
10	GSE4412-GPL96	Brain cancer	Glioma	UCLA (1996-2003)	Freije	HG-U133A	201353_s_at	74	0.189189	0.0099747	**-1.22433**	0,29 [0,12 - 0,75]
**BAZ2B**												
**Lp.**	**DATASET**	**CANCER TYPE**	**SUBTYPE**	**COHORT**	**AUTHOR**	**ARRAY TYPE**	**PROBE ID**	**N**	**CUTPOINT**	**COX P-VALUE**	**ln(HR)**	**HR [95% CI low - upp]**
**Worse prognosis**												
1	GSE9893	Breast cancer		Montpellier, Bordeaux, Turin (1989-2001)	Chanrion	MLRG Human 21K V12,0	13198	155	0.541936	0.0148604	**0.311118**	1,36 [1,06 - 1,75]
**Better prognosis**												
2	GSE4412-GPL96	Brain cancer	Glioma	UCLA (1996-2003)	Freije	HG-U133A	203080_s_at	74	0.378378	0.011085	**-0.635791**	0,53 [0,32 - 0,86]
3	GSE14764	Ovarian cancer		TOC	Denkert	HG-U133A	203080_s_at	80	0.15	0.0267242	**-0.956141**	0,38 [0,16 - 0,90]

4	jacob-00182-UM	Lung cancer	Adenocarcinoma	UM	Shedden	HG-U133A	203080_s_at	178	0.297753	0.0164622	**-1.12362**	0,33 [0,13 - 0,81]
5	GSE3143	Breast cancer		Duke	Bild	HG-U95A	37915_at	158	0.373418	0.000108984	**-1.74647**	0,17 [0,07 - 0,42]
6	GSE16581	Brain cancer	Meningioma	UCLA	Lee	HG-U133_Plus_2	203080_s_at	67	0.208955	0.0191139	**-2.9372**	0,05 [0,00 - 0,62]
7	GSE31210	Lung cancer	Adenocarcinoma	NCCRI	Okayama	HG-U133_Plus_2	203080_s_at	204	0.166667	0.000120679	**-3.25394**	0,04 [0,01 - 0,20]
**BPTF**												
**Lp.**	**DATASET**	**CANCER TYPE**	**SUBTYPE**	**COHORT**	**AUTHOR**	**ARRAY TYPE**	**PROBE ID**	**N**	**CUTPOINT**	**COX P-VALUE**	**ln(HR)**	**HR [95% CI low - upp]**
**Worse prognosis**												
1	GSE17537	Colorectal cancer		VMC	Smith	HG-U133_Plus_2	207186_s_at	55	0.8	0.00472897	**1.55723**	4,75 [1,61 - 13,98]
2	GSE19234	Skin cancer	Melanoma	NYU	Bogunovic	HG-U133_Plus_2	231953_at	38	0.263158	0.00320093	**1.38402**	3,99 [1,59 - 10,02]
3	GSE1456-GPL97	Breast cancer		Stockholm (1994-1996)	Pawitan	HG-U133B	231953_at	159	0.830189	0.0232726	**1.16215**	3,20 [1,17 - 8,72]
4	GSE3141	Lung cancer	NSCLC	Duke	Bild	HG-U133_Plus_2	231953_at	111	0.198198	0.0256529	**0.914027**	2,49 [1,12 - 5,57]
5	GSE13213	Lung cancer	Adenocarcinoma	Nagoya (1995-1999, 2002-2004)	Tomida	G4112F	A_23_P428548	117	0.82906	0.0168449	**0.80463**	2,24 [1,16 - 4,33]
6	GSE13213	Lung cancer	Adenocarcinoma	Nagoya (1995-1999, 2002-2004)	Tomida	G4112F	A_24_P392475	117	0.760684	0.0109845	**0.430486**	1,54 [1,10 - 2,14]
**Better prognosis**												
7	GSE4412-GPL97	Brain cancer	Glioma	UCLA (1996-2003)	Freije	HG-U133B	230056_at	74	0.472973	0.0405154	**-0.322838**	0,72 [0,53 - 0,99]
8	GSE4271-GPL97	Brain cancer	Astrocytoma	MDA	Phillips	HG-U133B	230056_at	77	0.662338	0.0235446	**-0.521206**	0,59 [0,38 - 0,93]
9	GSE4475	Blood cancer	B-cell lymphoma	Berlin (2003-2005)	Hummel	HG-U133A	209271_at	158	0.594937	0.0438516	**-0.571949**	0,56 [0,32 - 0,98]

10	jacob-00182-CANDF	Lung cancer	Adenocarcinoma	CAN/DF	Shedden	HG-U133A	207186_s_at	82	0.207317	0.0246746	**-0.808867**	0,45 [0,22 - 0,90]
11	GSE1456-GPL96	Breast cancer		Stockholm (1994-1996)	Pawitan	HG-U133A	207186_s_at	159	0.301887	0.0310216	**-0.816529**	0,44 [0,21 - 0,93]
12	jacob-00182-UM	Lung cancer	Adenocarcinoma	UM	Shedden	HG-U133A	207186_s_at	178	0.117978	0.00956273	**-0.824363**	0,44 [0,24 - 0,82]
13	GSE4412-GPL97	Brain cancer	Glioma	UCLA (1996-2003)	Freije	HG-U133B	232909_s_at	74	0.608108	0.011725	**-1.18007**	0,31 [0,12 - 0,77]
14	GSE4412-GPL96	Brain cancer	Glioma	UCLA (1996-2003)	Freije	HG-U133A	207186_s_at	74	0.77027	0.000865439	**-1.45904**	0,23 [0,10 - 0,55]
15	GSE31210	Lung cancer	Adenocarcinoma	NCCRI	Okayama	HG-U133_Plus_2	230056_at	204	0.416667	0.000576084	**-1.57136**	0,21 [0,08 - 0,51]
16	GSE4412-GPL96	Brain cancer	Glioma	UCLA (1996-2003)	Freije	HG-U133A	209271_at	74	0.783784	0.000470168	**-1.6612**	0,19 [0,07 - 0,48]
17	GSE31210	Lung cancer	Adenocarcinoma	NCCRI	Okayama	HG-U133_Plus_2	232909_s_at	204	0.480392	0.0193027	**-1.97924**	0,14 [0,03 - 0,73]
**BRD1**												
**Lp.**	**DATASET**	**CANCER TYPE**	**SUBTYPE**	**COHORT**	**AUTHOR**	**ARRAY TYPE**	**PROBE ID**	**N**	**CUTPOINT**	**COX P-VALUE**	**ln(HR)**	**HR [95% CI low - upp]**
**Worse prognosis**												
1	GSE8841	Ovarian cancer		Milan (1992-2003)	Marchini	G4100A	6815	81	0.48	0.0014	**2.38**	10,84 [2,51 - 46,76]
2	GSE9893	Breast cancer		Montpellier, Bordeaux, Turin (1989-2001)	Chanrion	MLRG Human 21K V12,0	6768	155	0.8	0.000341	**0.36**	1,43 [1,17 - 1,73]
**Better prognosis**												
3	DUKE-OC	Ovarian cancer		Duke	Bild	HG-U133A	204520_x_at	133	0.14	0.001627	**-0.43**	0,65 [0,50 - 0,85]
4	DUKE-OC	Ovarian cancer		Duke	Bild	HG-U133A	215460_x_at	133	0.14	0.002207	**-0.46**	0,63 [0,47 - 0,85]

5	GSE7390	Breast cancer		Uppsala, Oxford, Stockholm, IGR, GUYT, CRH (1980-1998)	Desmedt	HG-U133A	215460_x_at	198	0.26	0.044678	**-0.57**	0,57 [0,33 - 0,99]
6	GSE13213	Lung cancer	Adenocarcinoma	Nagoya (1995-1999, 2002-2004)	Tomida	G4112F	A_23_P166536	117	0.42	0.038162	**-0.64**	0,53 [0,29 - 0,97]
7	GSE7390	Breast cancer		Uppsala, Oxford, Stockholm, IGR, GUYT, CRH (1980-1998)	Desmedt	HG-U133A	204520_x_at	198	0.22	0.017157	**-0.71**	0,49 [0,28 - 0,88]
8	GSE4412-GPL96	Brain cancer	Glioma	UCLA (1996-2003)	Freije	HG-U133A	215460_x_at	74	0.16	0.025645	**-1.05**	0,35 [0,14 - 0,88]
9	jacob-00182-MSK	Lung cancer	Adenocarcinoma	MSK	Shedden	HG-U133A	204520_x_at	104	0.5	0.026435	**-1.29**	0,28 [0,09 - 0,86]
10	jacob-00182-MSK	Lung cancer	Adenocarcinoma	MSK	Shedden	HG-U133A	215460_x_at	104	0.51	0.028539	**-1.31**	0,27 [0,08 - 0,87]
11	GSE16581	Brain cancer	Meningioma	UCLA	Lee	HG-U133_Plus_2	204520_x_at	67	0.52	0.019845	**-3.33**	0,04 [0,00 - 0,59]
						**BRD2**						
**Lp.**	**DATASET**	**CANCER TYPE**	**SUBTYPE**	**COHORT**	**AUTHOR**	**ARRAY TYPE**	**PROBE ID**	**N**	**CUTPOINT**	**COX P-VALUE**	**ln(HR)**	**HR [95% CI low - upp]**
**Worse prognosis**												
1	E-TABM-346	Blood cancer	DLBCL	GELA (1998-2000)	Jais	HG-U133A	208685_x_at	53	0.830189	0.0189685	**1.86559**	6,46 [1,36 - 30,70]

2	E-TABM-346	Blood cancer	DLBCL	GELA (1998-2000)	Jais	HG-U133A	214911_s_at	53	0.867925	0.0416707	**1.58592**	4,88 [1,06 - 22,47]
3	jacob-00182-CANDF	Lung cancer	Adenocarcinoma	CAN/DF	Shedden	HG-U133A	208686_s_at	82	0.5	0.0211212	**1.49651**	4,47 [1,25 - 15,94]
4	GSE12945	Colorectal cancer		Berlin	Staub	HG-U133A	214911_s_at	62	0.887097	0.0365981	**1.1995**	2,74 [1,06 - 7,07]
5	GSE12945	Colorectal cancer		Berlin	Staub	HG-U133A	208685_x_at	62	0.887097	0.0375337	**1.09702**	3,00 [1,07 - 8,42]
**Better prognosis**												
6	GSE4412-GPL96	Brain cancer	Glioma	UCLA (1996-2003)	Freije	HG-U133A	208686_s_at	74	0.527027	0.0202397	**-1.01588**	0,36 [0,15 - 0,85]
7	jacob-00182-MSK	Lung cancer	Adenocarcinoma	MSK	Shedden	HG-U133A	214911_s_at	104	0.413462	0.0323903	**-1.21108**	0,30 [0,10 - 0,90]
8	jacob-00182-MSK	Lung cancer	Adenocarcinoma	MSK	Shedden	HG-U133A	208686_s_at	104	0.326923	0.00134581	**-2.70884**	0,07 [0,01 - 0,35]
**BRD3**												
**Lp.**	**DATASET**	**CANCER TYPE**	**SUBTYPE**	**COHORT**	**AUTHOR**	**ARRAY TYPE**	**PROBE ID**	**N**	**CUTPOINT**	**COX P-VALUE**	**ln(HR)**	**HR [95% CI low - upp]**
**Worse prognosis**												
1	E-TABM-346	Blood cancer	DLBCL	GELA (1998-2000)	Jais	HG-U133A	203825_at	53	0.849057	0.0475141	**1.393**	4,03 [1,02 - 15,97]
2	GSE5122	Blood cancer	AML	San Diego	Raponi	HG-U133A	203825_at	58	0.672414	0.049438	**0.839344**	2,31 [1,00 - 5,35]
3	GSE4475	Blood cancer	B-cell lymphoma	Berlin (2003-2005)	Hummel	HG-U133A	203825_at	158	0.221519	0.047073	**0.629683**	1,88 [1,01 - 3,49]
4	GSE3141	Lung cancer	NSCLC	Duke	Bild	HG-U133_Plus_2	1555028_at	111	0.576577	0.016514	**0.427156**	1,53 [1,08 - 2,17]
5	GSE7390	Breast cancer		Uppsala, Oxford, Stockholm, IGR, GUYT, CRH	Desmedt	HG-U133A	203825_at	198	0.479798	0.0390139	**0.424496**	1,53 [1,02 - 2,29]

				(1980-1998)								
6	GSE9893	Breast cancer		Montpellier, Bordeaux, Turin (1989-2001)	Chanrion	MLRG Human 21K V12,0	2570	155	0.83871	0.00754811	**0.247604**	1,28 [1,07 - 1,54]
**Better prognosis**												
7	GSE4412-GPL96	Brain cancer	Glioma	UCLA (1996-2003)	Freije	HG-U133A	203825_at	74	0.621622	0.0171521	**-0.93742**	0,39 [0,18 - 0,85]
8	GSE12417-GPL570	Blood cancer	AML	AMLCG (2004)	Metzeler	HG-U133_Plus_2	1555028_at	79	0.164557	0.0400344	**-1.8103**	0,16 [0,03 - 0,92]
**BRD4**												
**Lp.**	**DATASET**	**CANCER TYPE**	**SUBTYPE**	**COHORT**	**AUTHOR**	**ARRAY TYPE**	**PROBE ID**	**N**	**CUTPOINT**	**COX P-VALUE**	**ln(HR)**	**HR [95% CI low - upp]**
**Worse prognosis**												
1	GSE31210	Lung cancer	Adenocarcinoma	NCCRI	Okayama	HG-U133_Plus_2	202102_s_at	204	0.22549	0.00426889	**2.56502**	13,00 [2,24 - 75,52]
2	GSE8841	Ovarian cancer		Milan (1992-2003)	Marchini	G4100A	12779	81	0.740741	0.0295955	**1.50832**	4,52 [1,16 - 17,59]
3	GSE4412-GPL97	Brain cancer	Glioma	UCLA (1996-2003)	Freije	HG-U133B	239000_at	74	0.364865	0.0116901	**0.948422**	2,58 [1,24 - 5,40]
4	GSE13213	Lung cancer	Adenocarcinoma	Nagoya (1995-1999, 2002-2004)	Tomida	G4112F	A_23_P209011	117	0.846154	0.0382567	**0.529922**	1,70 [1,03 - 2,80]
5	DUKE-OC	Ovarian cancer		Duke	Bild	HG-U133A	202102_s_at	133	0.721804	0.000537735	**0.475691**	1,61 [1,23 - 2,11]
6	GSE4412-GPL97	Brain cancer	Glioma	UCLA (1996-2003)	Freije	HG-U133B	240360_at	74	0.783784	0.0180528	**0.467711**	1,60 [1,08 - 2,35]
**Better prognosis**												

7	GSE12417-GPL96	Blood cancer	AML	AMLCG (1999-2003)	Metzeler	HG-U133A	202103_at	163	0.858896	0.0336087	**-0.747514**	0,47 [0,24 - 0,94]
8	GSE19234	Skin cancer	Melanoma	NYU	Bogunovic	HG-U133_Plus_2	240360_at	38	0.184211	0.00722833	**-0.785309**	0,46 [0,26 - 0,81]
9	GSE8970	Blood cancer	AML	San Diego	Raponi	HG-U133A	202103_at	34	0.676471	0.0389772	**-0.939778**	0,39 [0,16 - 0,95]
10	GSE4412-GPL96	Brain cancer	Glioma	UCLA (1996-2003)	Freije	HG-U133A	202103_at	74	0.418919	0.011353	**-1.38647**	0,25 [0,09 - 0,73]
11	jacob-00182-UM	Lung cancer	Adenocarcinoma	UM	Shedden	HG-U133A	202102_s_at	178	0.224719	0.00152867	**-1.47706**	0,23 [0,09 - 0,57]
12	GSE12417-GPL570	Blood cancer	AML	AMLCG (2004)	Metzeler	HG-U133_Plus_2	240360_at	79	0.696203	0.0068142	**-2.48197**	0,08 [0,01 - 0,50]
**BRD7**												
**Lp.**	**DATASET**	**CANCER TYPE**	**SUBTYPE**	**COHORT**	**AUTHOR**	**ARRAY TYPE**	**PROBE ID**	**N**	**CUTPOINT**	**COX P-VALUE**	**ln(HR)**	**HR [95% CI low - upp]**
**Worse prognosis**												
1	GSE1456-GPL96	Breast cancer		Stockholm (1994-1996)	Pawitan	HG-U133A	221776_s_at	159	0.553459	0.0186357	**0.885307**	2,42 [1,16 - 5,07]
**Better prognosis**												
2	GSE4412-GPL97	Brain cancer	Glioma	UCLA (1996-2003)	Freije	HG-U133B	238545_at	74	0.108108	0.00658586	**-0.516904**	0,60 [0,41 - 0,87]
3	GSE9893	Breast cancer		Montpellier, Bordeaux, Turin (1989-2001)	Chanrion	MLRG Human 21K V12,0	13905	155	0.309677	0.000987744	**-0.63066**	0,53 [0,37 - 0,77]
4	GSE4271-GPL97	Brain cancer	Astrocytoma	MDA	Phillips	HG-U133B	238545_at	77	0.142857	0.016871	**-0.636611**	0,53 [0,31 - 0,89]
5	GSE4412-GPL97	Brain cancer	Glioma	UCLA (1996-2003)	Freije	HG-U133B	222737_s_at	74	0.310811	0.0131936	**-1.22475**	0,29 [0,11 - 0,77]
**BRD8**												

Lp.	DATASET	CANCER TYPE	SUBTYPE	COHORT	AUTHOR	ARRAY TYPE	PROBE ID	N	CUTP OINT	COX P-VALUE	ln(HR)	HR [95% CIlow - CIupp]
**Worse prognosis**												
1	DUKE-OC	Ovarian cancer		Duke	Bild	HG-U133A	210352_at	133	0.85	0.019433	**3.18**	23,96 [1,67 - 343,95]
2	GSE17536	Colorectal cancer		MCC	Smith	HG-U133_Plus_2	202227_s_at	177	0.53	0.004114	**1.18**	3,27 [1,46 - 7,34]
**Better prognosis**												
3	GSE31210	Lung cancer	Adenocarcinoma	NCCRI	Okayama	HG-U133_Plus_2	242265_at	204	0.12	0.024983	**-0.61**	0,54 [0,32 - 0,93]
4	GSE4412-GPL97	Brain cancer	Glioma	UCLA (1996-2003)	Freije	HG-U133B	242265_at	74	0.18	0.000286	**-0.74**	0,48 [0,32 - 0,71]
5	jacob-00182-UM	Lung cancer	Adenocarcinoma	UM	Shedden	HG-U133A	202227_s_at	178	0.13	0.00797	**-0.97**	0,38 [0,19 - 0,78]
6	jacob-00182-MSK	Lung cancer	Adenocarcinoma	MSK	Shedden	HG-U133A	202227_s_at	104	0.35	0.026805	**-1.18**	0,31 [0,11 - 0,87]
7	GSE17537	Colorectal cancer		VMC	Smith	HG-U133_Plus_2	242265_at	55	0.13	0.043559	**-2.46**	0,09 [0,01 - 0,93]
						**BRD9**						
**Lp.**	**DATASET**	**CANCER TYPE**	**SUBTYPE**	**COHORT**	**AUTHOR**	**ARRAY TYPE**	**PROBE ID**	**N**	**CUTP OINT**	**COX P-VALUE**	**ln(HR)**	**HR [95% CI low - upp]**
**Worse prognosis**												
1	GSE19234	Skin cancer	Melanoma	NYU	Bogunovic	HG-U133_Plus_2	220155_s_at	38	0.815789	0.033413	**1.09566**	2,99 [1,09 - 8,21]
2	GSE3141	Lung cancer	NSCLC	Duke	Bild	HG-U133_Plus_2	220155_s_at	111	0.486486	0.00150054	**1.03765**	2,82 [1,49 - 5,36]
3	GSE9891	Ovarian cancer		AOCS, RBH, WH, NKI-AVL (1992-2006)	Tothill	HG-U133_Plus_2	220155_s_at	278	0.492806	0.0000599246	**0.954315**	2,60 [1,63 - 4,14]
4	DUKE-OC	Ovarian cancer		Duke	Bild	HG-U133A	220155_s_at	133	0.203008	0.00536986	**0.597292**	1,82 [1,19 - 2,77]
**Better prognosis**												

Lp.	DATASET	CANCER TYPE	SUBTYPE	COHORT	AUTHOR	ARRAY TYPE	PROBE ID	N	CUTPOINT	COX P-VALUE	ln(HR)	HR [95% CI low - upp]
5	GSE11117	Lung cancer	NSCLC	Basel (2002-2005)	Baty	Novachip human 34,5k	H200015452	41	0.707317	0.0260597	**-0.747988**	0,47 [0,24 - 0,91]
6	GSE8841	Ovarian cancer		Milan (1992-2003)	Marchini	G4100A	8015	81	0.17284	0.0186557	**-1.65741**	0,19 [0,05 - 0,76]
7	GSE17537	Colorectal cancer		VMC	Smith	HG-U133_Plus_2	220155_s_at	55	0.254545	0.0364165	**-1.6883**	0,18 [0,04 - 0,90]
						**BRDT**						
**Lp.**	**DATASET**	**CANCER TYPE**	**SUBTYPE**	**COHORT**	**AUTHOR**	**ARRAY TYPE**	**PROBE ID**	**N**	**CUTPOINT**	**COX P-VALUE**	**ln(HR)**	**HR [95% CI low - upp]**
**Worse prognosis**												
1	E-TABM-158	Breast cancer		UCSF, CPMC (1989-1997)	Chin	HG-U133A	206787_at	117	0.641026	0.0026513	**2.29359**	9,91 [2,22 - 44,23]
2	GSE9891	Ovarian cancer		AOCS, RBH, WH, NKI-AVL (1992-2006)	Tothill	HG-U133_Plus_2	206787_at	278	0.568345	0.00385646	**0.576057**	1,78 [1,20 - 2,63]
3	DUKE-OC	Ovarian cancer		Duke	Bild	HG-U133A	206787_at	133	0.75188	0.0334782	**0.488486**	1,63 [1,04 - 2,56]
**Better prognosis**												
4	jacob-00182-MSK	Lung cancer	Adenocarcinoma	MSK	Shedden	HG-U133A	206787_at	104	0.817308	0.0421701	**-0.515803**	0,60 [0,36 - 0,98]
						**BRWD1**						
**Lp.**	**DATASET**	**CANCER TYPE**	**SUBTYPE**	**COHORT**	**AUTHOR**	**ARRAY TYPE**	**PROBE ID**	**N**	**CUTPOINT**	**COX P-VALUE**	**ln(HR)**	**HR [95% CI low - upp]**
**Worse prognosis**												
1	GSE12417-GPL570	Blood cancer	AML	AMLCG (2004)	Metzeler	HG-U133_Plus_2	244622_at	79	0.518987	0.0171358	**1.70367**	5,49 [1,35 - 22,30]
2	GSE11595	Esophagus cancer	Adenocarcinoma	Sutton	Giddings	CRUKDMF_22K_v1,0,0	814975	34	0.764706	0.0317395	**1.1594**	3,19 [1,11 - 9,18]
3	GSE12417-GPL97	Blood cancer	AML	AMLCG (1999-2003)	Metzeler	HG-U133B	231960_at	163	0.895706	0.0243889	**0.929157**	2,53 [1,13 - 5,69]

4	GSE13213	Lung cancer	Adenocarcinoma	Nagoya (1995-1999, 2002-2004)	Tomida	G4112F	A_24_P861009	117	0.418803	0.000613766	**0.790473**	2,20 [1,40 - 3,47]
5	GSE17536	Colorectal cancer		MCC	Smith	HG-U133_Plus_2	231960_at	177	0.502825	0.0345363	**0.749351**	2,12 [1,06 - 4,24]
6	GSE17536	Colorectal cancer		MCC	Smith	HG-U133_Plus_2	214820_at	177	0.548023	0.0432998	**0.504436**	1,66 [1,02 - 2,70]
7	GSE13213	Lung cancer	Adenocarcinoma	Nagoya (1995-1999, 2002-2004)	Tomida	G4112F	A_23_P211136	117	0.888889	0.0188484	**0.456454**	1,58 [1,08 - 2,31]
8	GSE5122	Blood cancer	AML	San Diego	Raponi	HG-U133A	219280_at	58	0.275862	0.0158012	**0.444804**	1,56 [1,09 - 2,24]
9	GSE12417-GPL97	Blood cancer	AML	AMLCG (1999-2003)	Metzeler	HG-U133B	225446_at	163	0.478528	0.013752	**0.436147**	1,55 [1,09 - 2,19]
**Better prognosis**												
10	GSE17260	Ovarian cancer		Niigata (1997-2008)	Yoshihara	G4112A	A_24_P231025	110	0.272727	0.0211222	**-0.272397**	0,76 [0,60 - 0,96]
11	GSE3141	Lung cancer	NSCLC	Duke	Bild	HG-U133_Plus_2	231960_at	111	0.468468	0.00625704	**-0.609819**	0,54 [0,35 - 0,84]
12	GSE4412-GPL97	Brain cancer	Glioma	UCLA (1996-2003)	Freije	HG-U133B	231960_at	74	0.135135	0.0153697	**-0.796771**	0,45 [0,24 - 0,86]
13	GSE4412-GPL96	Brain cancer	Glioma	UCLA (1996-2003)	Freije	HG-U133A	214820_at	74	0.621622	0.00255224	**-0.805348**	0,45 [0,26 - 0,75]
14	GSE31210	Lung cancer	Adenocarcinoma	NCCRI	Okayama	HG-U133_Plus_2	214820_at	204	0.387255	0.00355505	**-1.52493**	0,22 [0,08 - 0,61]
15	GSE31210	Lung cancer	Adenocarcinoma	NCCRI	Okayama	HG-U133_Plus_2	231960_at	204	0.127451	0.000526576	**-1.53489**	0,22 [0,09 - 0,51]
16	GSE31210	Lung cancer	Adenocarcinoma	NCCRI	Okayama	HG-U133_Plus_2	1553227_s_at	204	0.161765	0.012318	**-1.5359**	0,22 [0,06 - 0,72]
**BRPF1**												
**Lp.**	**DATASET**	**CANCER TYPE**	**SUBTYPE**	**COHORT**	**AUTHOR**	**ARRAY TYPE**	**PROBE ID**	**N**	**CUTPOINT**	**COX P-VALUE**	**ln(HR)**	**HR [95% CI low - upp]**

**Better prognosis**												
1	GSE4412-GPL96	Brain cancer	Glioma	UCLA (1996-2003)	Freije	HG-U133A	204481_at	74	0.22	0.043995	**-0.97**	0,38 [0,15 - 0,97]
**BRPF3**												
**Lp.**	**DATASET**	**CANCER TYPE**	**SUBTYPE**	**COHORT**	**AUTHOR**	**ARRAY TYPE**	**PROBE ID**	**N**	**CUTP OINT**	**COX P-VALUE**	**ln(HR)**	**HR [95% CI low - upp]**
**Worse prognosis**												
1	GSE13213	Lung cancer	Adenocarcinoma	Nagoya (1995-1999, 2002-2004)	Tomida	G4112F	A_24_P4147 12	117	0.49	0.006187	**0.62**	1,87 [1,19 - 2,92]
2	GSE9893	Breast cancer		Montpellier, Bordeaux, Turin (1989-2001)	Chanrion	MLRG Human 21K V12,0	12540	155	0.85	0.000209	**0.32**	1,37 [1,16 - 1,63]
**BRWD3**												
**Lp.**	**DATASET**	**CANCER TYPE**	**SUBTYPE**	**COHORT**	**AUTHOR**	**ARRAY TYPE**	**PROBE ID**	**N**	**CUTP OINT**	**COX P-VALUE**	**ln(HR)**	**HR [95% CI low - upp]**
**Worse prognosis**												
1	GSE13213	Lung cancer	Adenocarcinoma	Nagoya (1995-1999, 2002-2004)	Tomida	G4112F	A_32_P4891 30	117	0.5470 09	0.003505 51	**0.651056**	1,92 [1,24 - 2,97]
2	GSE16131 -GPL97	Blood cancer	Follicular lymphoma	NCI (1974-2001)	Dave	HG-U133B	244738_at	180	0.3277 78	0.043932 6	**0.553631**	1,74 [1,02 - 2,98]
**CECR2**												
**Lp.**	**DATASET**	**CANCER TYPE**	**SUBTYPE**	**COHORT**	**AUTHOR**	**ARRAY TYPE**	**PROBE ID**	**N**	**CUTP OINT**	**COX P-VALUE**	**ln(HR)**	**HR [95% CI low - upp]**
**Better prognosis**												
1	GSE13213	Lung cancer	Adenocarcinoma	Nagoya (1995-1999,	Tomida	G4112F	A_23_P2113 26	117	0.2307 69	0.002209 5	**-0.641039**	0,53 [0,35 - 0,79]

				2002-2004)								
2	GSE31210	Lung cancer	Adenocarcinoma	NCCRI	Okayama	HG-U133_Plus_2	223729_at	204	0.142157	0.00202362	**-1.00205**	0,37 [0,19 - 0,69]
3	GSE17536	Colorectal cancer		MCC	Smith	HG-U133_Plus_2	233695_s_at	177	0.225989	0.0347797	**-1.24088**	0,29 [0,09 - 0,92]
4	GSE12417-GPL570	Blood cancer	AML	AMLCG (2004)	Metzeler	HG-U133_Plus_2	233695_s_at	79	0.291139	0.00245849	**-3.79271**	0,02 [0,00 - 0,26]
						**EP300**						
**Lp.**	**DATASET**	**CANCER TYPE**	**SUBTYPE**	**COHORT**	**AUTHOR**	**ARRAY TYPE**	**PROBE ID**	**N**	**CUTPOINT**	**COX P-VALUE**	**ln(HR)**	**HR [95% CI low - upp]**
**Worse prognosis**												
1	GSE17537	Colorectal cancer		VMC	Smith	HG-U133_Plus_2	213579_s_at	55	0.55	0.048223	**0.69**	1,99 [1,01 - 3,93]
**Better prognosis**												
2	GSE12417-GPL96	Blood cancer	AML	AMLCG (1999-2003)	Metzeler	HG-U133A	213579_s_at	163	0.67	0.038678	**-0.4**	0,67 [0,46 - 0,98]
3	GSE3143	Breast cancer		Duke	Bild	HG-U95A	33896_at	158	0.33	0.007682	**-0.61**	0,54 [0,35 - 0,85]
4	jacob-00182-UM	Lung cancer	Adenocarcinoma	UM	Shedden	HG-U133A	213579_s_at	178	0.16	0.04784	**-0.7**	0,49 [0,25 - 0,99]
5	MICHIGAN-LC	Lung cancer	Adenocarcinoma	Michigan (1994-2000)	Beer	HuGeneFL	U01877_at	86	0.27	0.031454	**-0.79**	0,45 [0,22 - 0,93]
6	MGH-glioma	Brain cancer	Glioma	CBTTB, MGH, BWH, CH	Nutt	HG-U95A	33896_at	50	0.54	0.036549	**-1.75**	0,17 [0,03 - 0,90]
7	GSE31210	Lung cancer	Adenocarcinoma	NCCRI	Okayama	HG-U133_Plus_2	213579_s_at	204	0.27	0.000236	**-3.15**	0,04 [0,01 - 0,23]
						**KAT2A**						
**Lp.**	**DATASET**	**CANCER TYPE**	**SUBTYPE**	**COHORT**	**AUTHOR**	**ARRAY TYPE**	**PROBE ID**	**N**	**CUTPOINT**	**COX P-VALUE**	**ln(HR)**	**HR [95% CI low - upp]**
**Worse prognosis**												
1	GSE8841	Ovarian cancer		Milan (1992-2003)	Marchini	G4100A	9377	81	0.83	0.005305	**2.21**	9,14 [1,93 - 43,33]

2	GSE8841	Ovarian cancer		Milan (1992-2003)	Marchini	G4100A	2419	81	0.81	0.003046	**1.41**	4,09 [1,61 - 10,38]
3	GSE19234	Skin cancer	Melanoma	NYU	Bogunovic	HG-U133_Plus_2	202182_at	38	0.66	0.039245	**1.16**	3,18 [1,06 - 9,57]
4	GSE12417-GPL96	Blood cancer	AML	AMLCG (1999-2003)	Metzeler	HG-U133A	202182_at	163	0.64	0.003786	**0.79**	2,20 [1,29 - 3,74]
**Better prognosis**												
5	GSE4412-GPL96	Brain cancer	Glioma	UCLA (1996-2003)	Freije	HG-U133A	202182_at	74	0.12	0.000839	**-1.68**	0,19 [0,07 - 0,50]
6	GSE31210	Lung cancer	Adenocarcinoma	NCCRI	Okayama	HG-U133_Plus_2	202182_at	204	0.57	0.006382	**-1.72**	0,18 [0,05 - 0,62]
**KMT2A**												
**Lp.**	**DATASET**	**CANCER TYPE**	**SUBTYPE**	**COHORT**	**AUTHOR**	**ARRAY TYPE**	**PROBE ID**	**N**	**CUTPOINT**	**COX P-VALUE**	**ln(HR)**	**HR [95% CI low - upp]**
**Worse prognosis**												
1	GSE8841	Ovarian cancer		Milan (1992-2003)	Marchini	G4100A	9377	81	0.83	0.005305	**2.21**	9,14 [1,93 - 43,33]
2	GSE8841	Ovarian cancer		Milan (1992-2003)	Marchini	G4100A	2419	81	0.81	0.003046	**1.41**	4,09 [1,61 - 10,38]
3	GSE19234	Skin cancer	Melanoma	NYU	Bogunovic	HG-U133_Plus_2	202182_at	38	0.66	0.039245	**1.16**	3,18 [1,06 - 9,57]
4	GSE12417-GPL96	Blood cancer	AML	AMLCG (1999-2003)	Metzeler	HG-U133A	202182_at	163	0.64	0.003786	**0.79**	2,20 [1,29 - 3,74]
**Better prognosis**												
5	GSE4412-GPL96	Brain cancer	Glioma	UCLA (1996-2003)	Freije	HG-U133A	202182_at	74	0.12	0.000839	**-1.68**	0,19 [0,07 - 0,50]
6	GSE31210	Lung cancer	Adenocarcinoma	NCCRI	Okayama	HG-U133_Plus_2	202182_at	204	0.57	0.006382	**-1.72**	0,18 [0,05 - 0,62]
**PBRM1**												
**Lp.**	**DATASET**	**CANCER TYPE**	**SUBTYPE**	**COHORT**	**AUTHOR**	**ARRAY TYPE**	**PROBE ID**	**N**	**CUTPOINT**	**COX P-VALUE**	**ln(HR)**	**HR [95% CI low - upp]**

**Worse prognosis**												
1	GSE13213	Lung cancer	Adenocarcinoma	Nagoya (1995-1999, 2002-2004)	Tomida	G4112F	A_23_P365874	117	0.871795	0.00578988	**0.89099**	2,44 [1,29 - 4,59]
2	GSE13213	Lung cancer	Adenocarcinoma	Nagoya (1995-1999, 2002-2004)	Tomida	G4112F	A_23_P218863	117	0.837607	0.000324076	**0.64465**	1,91 [1,34 - 2,71]
3	DUKE-OC	Ovarian cancer		Duke	Bild	HG-U133A	221212_x_at	133	0.676692	0.0118556	**0.393**	1,48 [1,09 - 2,01]
**Better prognosis**												
4	DUKE-OC	Ovarian cancer		Duke	Bild	HG-U133A	220355_s_at	133	0.699248	0.0238351	**-0.327242**	0,72 [0,54 - 0,96]
5	GSE1456-GPL97	Breast cancer		Stockholm (1994-1996)	Pawitan	HG-U133B	223399_x_at	159	0.138365	0.0458298	**-0.481372**	0,62 [0,39 - 0,99]
6	GSE4412-GPL97	Brain cancer	Glioma	UCLA (1996-2003)	Freije	HG-U133B	223400_s_at	74	0.675676	0.0199856	**-0.484632**	0,62 [0,41 - 0,93]
7	jacob-00182-MSK	Lung cancer	Adenocarcinoma	MSK	Shedden	HG-U133A	220355_s_at	104	0.201923	0.0190315	**-1.65229**	0,19 [0,05 - 0,76]
8	GSE12417-GPL570	Blood cancer	AML	AMLCG (2004)	Metzeler	HG-U133_Plus_2	223399_x_at	79	0.556962	0.0142038	**-2.34628**	0,10 [0,01 - 0,62]
9	GSE17537	Colorectal cancer		VMC	Smith	HG-U133_Plus_2	223899_at	55	0.327273	0.000505917	**-3.77682**	0,02 [0,00 - 0,19]
						**PHIP**						
**Lp.**	**DATASET**	**CANCER TYPE**	**SUBTYPE**	**COHORT**	**AUTHOR**	**ARRAY TYPE**	**PROBE ID**	**N**	**CUTPOINT**	**COX P-VALUE**	**ln(HR)**	**HR [95% CI low - upp]**
**Worse prognosis**												
1	GSE17537	Colorectal cancer		VMC	Smith	HG-U133_Plus_2	212542_s_at	55	0.672727	0.0221034	**1.03431**	2,81 [1,16 - 6,82]
2	GSE17536	Colorectal cancer		MCC	Smith	HG-U133_Plus_2	212542_s_at	177	0.485876	0.020837	**0.798699**	2,22 [1,13 - 4,38]
**Better prognosis**												

3	GSE16131-GPL96	Blood cancer	Follicular lymphoma	NCI (1974-2001)	Dave	HG-U133A	212542_s_at	180	0.122222	0.0129036	**-0.521375**	0,59 [0,39 - 0,90]
4	GSE5287	Bladder cancer		Aarhus (1995-2004)	Als	HG-U133A	212542_s_at	30	0.7	0.0388037	**-0.734735**	0,48 [0,24 - 0,96]
5	GSE31210	Lung cancer	Adenocarcinoma	NCCRI	Okayama	HG-U133_Plus_2	212542_s_at	204	0.411765	0.0128837	**-1.51592**	0,22 [0,07 - 0,73]
**SMARCA2**												
**Lp.**	**DATASET**	**CANCER TYPE**	**SUBTYPE**	**COHORT**	**AUTHOR**	**ARRAY TYPE**	**PROBE ID**	**N**	**CUTPOINT**	**COX P-VALUE**	**ln(HR)**	**HR [95% CI low - upp]**
**Worse prognosis**												
1	DUKE-OC	Ovarian cancer		Duke	Bild	HG-U133A	212258_s_at	133	0.66	0.012519	**2.8**	16,43 [1,83 - 147,90]
2	GSE17537	Colorectal cancer		VMC	Smith	HG-U133_Plus_2	206543_at	55	0.87	0.00003	**2.37**	10,71 [3,52 - 32,58]
3	GSE8841	Ovarian cancer		Milan (1992-2003)	Marchini	G4100A	8609	81	0.84	0.014528	**0.85**	2,35 [1,18 - 4,65]
4	GSE17536	Colorectal cancer		MCC	Smith	HG-U133_Plus_2	206542_s_at	177	0.7	0.023334	**0.66**	1,93 [1,09 - 3,41]
5	GSE17536	Colorectal cancer		MCC	Smith	HG-U133_Plus_2	206543_at	177	0.53	0.0093	**0.65**	1,91 [1,17 - 3,11]
6	GSE5122	Blood cancer	AML	San Diego	Raponi	HG-U133A	212257_s_at	58	0.43	0.02979	**0.59**	1,80 [1,06 - 3,07]
7	GSE7696	Brain cancer	Glioblastoma	Lausanne	Murat	HG-U133_Plus_2	206542_s_at	70	0.16	0.043869	**0.38**	1,47 [1,01 - 2,13]
8	GSE9893	Breast cancer		Montpellier, Bordeaux, Turin (1989-2001)	Chanrion	MLRG Human 21K V12,0	13420	155	0.85	0.040565	**0.17**	1,19 [1,01 - 1,39]
**Better prognosis**												
9	DUKE-OC	Ovarian cancer		Duke	Bild	HG-U133A	206544_x_at	133	0.12	0.001172	**-0.27**	0,77 [0,65 - 0,90]
10	DUKE-OC	Ovarian cancer		Duke	Bild	HG-U133A	206542_s_at	133	0.55	0.001379	**-0.33**	0,72 [0,59 - 0,88]
11	jacob-00182-CANDF	Lung cancer	Adenocarcinoma	CAN/DF	Shedden	HG-U133A	206544_x_at	82	0.11	0.049924	**-0.54**	0,58 [0,34 - 1,00]

12	GSE1456-GPL96	Breast cancer		Stockholm (1994-1996)	Pawitan	HG-U133A	206542_s_at	159	0.33	0.009689	**-0.62**	0,54 [0,33 - 0,86]
13	jacob-00182-CANDF	Lung cancer	Adenocarcinoma	CAN/DF	Shedden	HG-U133A	206542_s_at	82	0.13	0.02312	**-0.66**	0,52 [0,29 - 0,91]
14	GSE14814	Lung cancer	NSCLC	JRB,10	Zhu	HG-U133A	212257_s_at	90	0.67	0.046407	**-0.78**	0,46 [0,21 - 0,99]
15	GSE8841	Ovarian cancer		Milan (1992-2003)	Marchini	G4100A	15274	81	0.27	0.018453	**-0.8**	0,45 [0,23 - 0,87]
16	jacob-00182-MSK	Lung cancer	Adenocarcinoma	MSK	Shedden	HG-U133A	206543_at	104	0.18	0.038991	**-0.88**	0,41 [0,18 - 0,96]
17	GSE3143	Breast cancer		Duke	Bild	HG-U95A	40961_at	158	0.14	0.004004	**-0.97**	0,38 [0,20 - 0,73]
18	jacob-00182-CANDF	Lung cancer	Adenocarcinoma	CAN/DF	Shedden	HG-U133A	212257_s_at	82	0.46	0.008446	**-1.28**	0,28 [0,11 - 0,72]
19	GSE9891	Ovarian cancer		AOCS, RBH, WH, NKI-AVL (1992-2006)	Tothill	HG-U133_Plus_2	228926_s_at	278	0.86	0.017762	**-1.36**	0,26 [0,08 - 0,79]
20	GSE31210	Lung cancer	Adenocarcinoma	NCCRI	Okayama	HG-U133_Plus_2	206542_s_at	204	0.28	0.000416	**-1.72**	0,18 [0,07 - 0,47]
						**SMARCA4**						
**Lp.**	**DATASET**	**CANCER TYPE**	**SUBTYPE**	**COHORT**	**AUTHOR**	**ARRAY TYPE**	**PROBE ID**	**N**	**CUTPOINT**	**COX P-VALUE**	**ln(HR)**	**HR [95% CI low - upp]**
**Worse prognosis**												
1	DUKE-OC	Ovarian cancer		Duke	Bild	HG-U133A	213719_s_at	133	0.65	0.018297	**3.56**	35,02 [1,83 - 671,61]
2	DUKE-OC	Ovarian cancer		Duke	Bild	HG-U133A	214360_at	133	0.63	0.00795	**3.36**	28,69 [2,41 - 342,22]
3	GSE1456-GPL96	Breast cancer		Stockholm (1994-1996)	Pawitan	HG-U133A	208793_x_at	159	0.5	0.000719	**1.44**	4,21 [1,83 - 9,69]
4	jacob-00182-CANDF	Lung cancer	Adenocarcinoma	CAN/DF	Shedden	HG-U133A	213719_s_at	82	0.82	0.021784	**1.11**	3,04 [1,18 - 7,86]

5	GSE1456-GPL96	Breast cancer		Stockholm (1994-1996)	Pawitan	HG-U133A	208794_s_at	159	0.86	0.004726	**1.09**	2,96 [1,39 - 6,30]
6	GSE1456-GPL96	Breast cancer		Stockholm (1994-1996)	Pawitan	HG-U133A	215714_s_at	159	0.48	0.006765	**1.03**	2,79 [1,33 - 5,88]
7	GSE1456-GPL96	Breast cancer		Stockholm (1994-1996)	Pawitan	HG-U133A	214728_x_at	159	0.9	0.006899	**0.99**	2,70 [1,31 - 5,54]
8	GSE9893	Breast cancer		Montpellier, Bordeaux, Turin (1989-2001)	Chanrion	MLRG Human 21K V12,0	15678	155	0.7	0.004046	**0.37**	1,45 [1,12 - 1,86]
9	DUKE-OC	Ovarian cancer		Duke	Bild	HG-U133A	214728_x_at	133	0.79	0.022657	**0.27**	1,31 [1,04 - 1,66]
10	DUKE-OC	Ovarian cancer		Duke	Bild	HG-U133A	208794_s_at	133	0.5	0.038999	**0.27**	1,30 [1,01 - 1,68]
11	DUKE-OC	Ovarian cancer		Duke	Bild	HG-U133A	212520_s_at	133	0.44	0.008166	**0.25**	1,28 [1,07 - 1,54]
**Better prognosis**												
12	GSE17260	Ovarian cancer		Niigata (1997-2008)	Yoshihara	G4112A	A_24_P212851	110	0.12	0.040482	**-0.31**	0,73 [0,54 - 0,99]
13	GSE4475	Blood cancer	B-cell lymphoma	Berlin (2003-2005)	Hummel	HG-U133A	212520_s_at	158	0.82	0.028301	**-0.33**	0,72 [0,53 - 0,97]
14	GSE4475	Blood cancer	B-cell lymphoma	Berlin (2003-2005)	Hummel	HG-U133A	215714_s_at	158	0.34	0.014319	**-0.39**	0,68 [0,50 - 0,93]
15	GSE4475	Blood cancer	B-cell lymphoma	Berlin (2003-2005)	Hummel	HG-U133A	213720_s_at	158	0.81	0.005445	**-0.42**	0,66 [0,49 - 0,88]
16	GSE4475	Blood cancer	B-cell lymphoma	Berlin (2003-2005)	Hummel	HG-U133A	208794_s_at	158	0.13	0.003155	**-0.42**	0,66 [0,50 - 0,87]
17	jacob-00182-UM	Lung cancer	Adenocarcinoma	UM	Shedden	HG-U133A	208794_s_at	178	0.16	0.040504	**-0.42**	0,66 [0,44 - 0,98]
18	GSE4475	Blood cancer	B-cell lymphoma	Berlin (2003-2005)	Hummel	HG-U133A	214728_x_at	158	0.82	0.006845	**-0.44**	0,65 [0,47 - 0,89]

19	GSE5122	Blood cancer	AML	San Diego	Raponi	HG-U133A	208794_s_at	58	0.45	0.045994	**-0.46**	0,63 [0,41 - 0,99]
20	GSE4475	Blood cancer	B-cell lymphoma	Berlin (2003-2005)	Hummel	HG-U133A	208793_x_at	158	0.49	0.02102	**-0.48**	0,62 [0,41 - 0,93]
21	jacob-00182-UM	Lung cancer	Adenocarcinoma	UM	Shedden	HG-U133A	212520_s_at	178	0.25	0.031141	**-0.48**	0,62 [0,40 - 0,96]
22	GSE1456-GPL96	Breast cancer		Stockholm (1994-1996)	Pawitan	HG-U133A	214360_at	159	0.36	0.014972	**-0.5**	0,61 [0,40 - 0,91]
23	GSE14764	Ovarian cancer		TOC	Denkert	HG-U133A	215714_s_at	80	0.16	0.006882	**-0.5**	0,60 [0,42 - 0,87]
24	jacob-00182-UM	Lung cancer	Adenocarcinoma	UM	Shedden	HG-U133A	215714_s_at	178	0.22	0.015329	**-0.53**	0,59 [0,38 - 0,90]
25	jacob-00182-UM	Lung cancer	Adenocarcinoma	UM	Shedden	HG-U133A	213720_s_at	178	0.24	0.033926	**-0.55**	0,57 [0,34 - 0,96]
26	jacob-00182-UM	Lung cancer	Adenocarcinoma	UM	Shedden	HG-U133A	214728_x_at	178	0.2	0.048105	**-0.59**	0,55 [0,31 - 1,00]
27	jacob-00182-HLM	Lung cancer	Adenocarcinoma	HLM	Shedden	HG-U133A	208794_s_at	79	0.32	0.031576	**-0.61**	0,54 [0,31 - 0,95]
28	GSE14764	Ovarian cancer		TOC	Denkert	HG-U133A	214728_x_at	80	0.15	0.031674	**-0.69**	0,50 [0,27 - 0,94]
29	jacob-00182-HLM	Lung cancer	Adenocarcinoma	HLM	Shedden	HG-U133A	212520_s_at	79	0.3	0.028697	**-0.7**	0,50 [0,27 - 0,93]
30	GSE14764	Ovarian cancer		TOC	Denkert	HG-U133A	208794_s_at	80	0.15	0.025307	**-0.72**	0,49 [0,26 - 0,92]
31	GSE4412-GPL96	Brain cancer	Glioma	UCLA (1996-2003)	Freije	HG-U133A	208793_x_at	74	0.15	0.002215	**-0.74**	0,48 [0,30 - 0,77]
32	GSE4412-GPL96	Brain cancer	Glioma	UCLA (1996-2003)	Freije	HG-U133A	213720_s_at	74	0.11	0.033761	**-0.77**	0,46 [0,23 - 0,94]
33	GSE17536	Colorectal cancer		MCC	Smith	HG-U133_Plus_2	214360_at	177	0.79	0.028261	**-1.23**	0,29 [0,10 - 0,88]
34	GSE4412-GPL96	Brain cancer	Glioma	UCLA (1996-2003)	Freije	HG-U133A	215714_s_at	74	0.22	0.01174	**-1.31**	0,27 [0,10 - 0,75]
35	GSE31210	Lung cancer	Adenocarcinoma	NCCRI	Okayama	HG-U133_Plus_2	1569073_x_at	204	0.18	0.006571	**-1.51**	0,22 [0,07 - 0,66]

Lp.	DATASET	CANCER TYPE	SUBTYPE	COHORT	AUTHOR	ARRAY TYPE	PROBE ID	N	CUTPOINT	COX P-VALUE	ln(HR)	HR [95% CI low - upp]
36	GSE12417-GPL96	Blood cancer	AML	AMLCG (1999-2003)	Metzeler	HG-U133A	214360_at	163	0.19	0.022784	**-1.53**	0,22 [0,06 - 0,81]
37	GSE12417-GPL570	Blood cancer	AML	AMLCG (2004)	Metzeler	HG-U133_Plus_2	214360_at	79	0.16	0.006666	**-2.34**	0,10 [0,02 - 0,52]
38	GSE12417-GPL570	Blood cancer	AML	AMLCG (2004)	Metzeler	HG-U133_Plus_2	213719_s_at	79	0.11	0.007002	**-2.73**	0,07 [0,01 - 0,47]

SP100

Lp.	DATASET	CANCER TYPE	SUBTYPE	COHORT	AUTHOR	ARRAY TYPE	PROBE ID	N	CUTPOINT	COX P-VALUE	ln(HR)	HR [95% CI low - upp]
**Worse prognosis**												
1	GSE31210	Lung cancer	Adenocarcinoma	NCCRI	Okayama	HG-U133_Plus_2	202864_s_at	204	0.897059	0.0220605	**1.78176**	5,94 [1,29 - 27,31]
2	GSE31210	Lung cancer	Adenocarcinoma	NCCRI	Okayama	HG-U133_Plus_2	210218_s_at	204	0.632353	0.00852057	**1.57048**	4,81 [1,49 - 15,50]
3	GSE31210	Lung cancer	Adenocarcinoma	NCCRI	Okayama	HG-U133_Plus_2	210219_at	204	0.583333	0.00245573	**1.51392**	4,54 [1,71 - 12,11]
4	E-TABM-346	Blood cancer	DLBCL	GELA (1998-2000)	Jais	HG-U133A	202864_s_at	53	0.867925	0.00537402	**1.26299**	3,54 [1,45 - 8,60]
5	jacob-00182-MSK	Lung cancer	Adenocarcinoma	MSK	Shedden	HG-U133A	210218_s_at	104	0.567308	0.0466377	**0.912667**	2,49 [1,01 - 6,12]
6	GSE7696	Brain cancer	Glioblastoma	Lausanne	Murat	HG-U133_Plus_2	237426_at	70	0.442857	0.0438762	**0.810681**	2,25 [1,02 - 4,95]
7	GSE17537	Colorectal cancer		VMC	Smith	HG-U133_Plus_2	202863_at	55	0.545455	0.0161217	**0.802328**	2,23 [1,16 - 4,29]
8	GSE12417-GPL570	Blood cancer	AML	AMLCG (2004)	Metzeler	HG-U133_Plus_2	202864_s_at	79	0.835443	0.0347013	**0.546137**	1,73 [1,04 - 2,87]
9	MGH-glioma	Brain cancer	Glioma	CBTTB, MGH, BWH, CH	Nutt	HG-U95A	37353_g_at	50	0.64	0.016096	**0.493601**	1,64 [1,10 - 2,45]
10	GSE4412-GPL96	Brain cancer	Glioma	UCLA (1996-2003)	Freije	HG-U133A	202863_at	74	0.459459	0.0409634	**0.471637**	1,60 [1,02 - 2,52]
**Better prognosis**												
11	GSE16560	Prostate cancer		Sweden (1977-1999)	Sboner	6K DASL	DAP4_2216	281	0.530249	0.0497672	**-0.26071**	0,77 [0,59 - 1,00]

12	DUKE-OC	Ovarian cancer		Duke	Bild	HG-U133A	202863_at	133	0.105263	0.0357698	**-0.269483**	0,76 [0,59 - 0,98]
13	GSE5122	Blood cancer	AML	San Diego	Raponi	HG-U133A	202863_at	58	0.344828	0.0275151	**-0.434624**	0,65 [0,44 - 0,95]
14	GSE9893	Breast cancer		Montpellier, Bordeaux, Turin (1989-2001)	Chanrion	MLRG Human 21K V12,0	6493	155	0.406452	0.00101127	**-0.476229**	0,62 [0,47 - 0,83]
15	GSE1456-GPL97	Breast cancer		Stockholm (1994-1996)	Pawitan	HG-U133B	237426_at	159	0.339623	0.00476704	**-0.555122**	0,57 [0,39 - 0,84]
16	GSE26712	Ovarian cancer		MSKCC (1990-2003)	Bonome	HG-U133_Plus_2	210218_s_at	185	0.810811	0.0173369	**-0.577126**	0,56 [0,35 - 0,90]
17	GSE13213	Lung cancer	Adenocarcinoma	Nagoya (1995-1999, 2002-2004)	Tomida	G4112F	A_23_P209712	117	0.205128	0.0194764	**-0.668882**	0,51 [0,29 - 0,90]
18	GSE1456-GPL96	Breast cancer		Stockholm (1994-1996)	Pawitan	HG-U133A	202864_s_at	159	0.245283	0.0217924	**-0.921791**	0,40 [0,18 - 0,87]
19	GSE31210	Lung cancer	Adenocarcinoma	NCCRI	Okayama	HG-U133_Plus_2	237426_at	204	0.102941	0.0029019	**-1.20212**	0,30 [0,14 - 0,66]
20	GSE13213	Lung cancer	Adenocarcinoma	Nagoya (1995-1999, 2002-2004)	Tomida	G4112F	A_24_P915158	117	0.324786	0.0000127868	**-1.45506**	0,23 [0,12 - 0,45]
						**SP110**						
**Lp.**	**DATASET**	**CANCER TYPE**	**SUBTYPE**	**COHORT**	**AUTHOR**	**ARRAY TYPE**	**PROBE ID**	**N**	**CUTPOINT**	**COX P-VALUE**	**ln(HR)**	**HR [95% CI low - upp]**
**Worse prognosis**												
1	GSE17537	Colorectal cancer		VMC	Smith	HG-U133_Plus_2	208392_x_at	55	0.581818	0.00754612	**2.36007**	10,59 [1,88 - 59,83]
2	GSE4412-GPL96	Brain cancer	Glioma	UCLA (1996-2003)	Freije	HG-U133A	209762_x_at	74	0.72973	0.00970211	**1.36273**	3,91 [1,39 - 10,97]

3	GSE31210	Lung cancer	Adenocarcinoma	NCCRI	Okayama	HG-U133_Plus_2	208012_x_at	204	0.720588	0.00630618	**1.32681**	3,77 [1,45 - 9,77]
4	GSE31210	Lung cancer	Adenocarcinoma	NCCRI	Okayama	HG-U133_Plus_2	209761_s_at	204	0.519608	0.0206997	**1.15577**	3,18 [1,19 - 8,46]
5	GSE4412-GPL96	Brain cancer	Glioma	UCLA (1996-2003)	Freije	HG-U133A	208012_x_at	74	0.662162	0.002955	**1.08993**	2,97 [1,45 - 6,10]
6	GSE31210	Lung cancer	Adenocarcinoma	NCCRI	Okayama	HG-U133_Plus_2	209762_x_at	204	0.862745	0.022698	**1.06159**	2,89 [1,16 - 7,21]
7	GSE4475	Blood cancer	B-cell lymphoma	Berlin (2003-2005)	Hummel	HG-U133A	208392_x_at	158	0.170886	0.00511553	**0.964396**	2,62 [1,34 - 5,15]
8	GSE4271-GPL96	Brain cancer	Astrocytoma	MDA	Phillips	HG-U133A	208012_x_at	77	0.311688	0.045646	**0.553302**	1,74 [1,01 - 2,99]
9	GSE4475	Blood cancer	B-cell lymphoma	Berlin (2003-2005)	Hummel	HG-U133A	209761_s_at	158	0.392405	0.0169763	**0.534775**	1,71 [1,10 - 2,65]
**Better prognosis**												
10	GSE19234	Skin cancer	Melanoma	NYU	Bogunovic	HG-U133_Plus_2	208392_x_at	38	0.368421	0.00159837	**-1.22662**	0,29 [0,14 - 0,63]
11	GSE19234	Skin cancer	Melanoma	NYU	Bogunovic	HG-U133_Plus_2	223980_s_at	38	0.105263	0.000407086	**-1.49843**	0,22 [0,10 - 0,51]
						**SP140**						
**Lp.**	**DATASET**	**CANCER TYPE**	**SUBTYPE**	**COHORT**	**AUTHOR**	**ARRAY TYPE**	**PROBE ID**	**N**	**CUTPOINT**	**COX P-VALUE**	**ln(HR)**	**HR [95% CI low - upp]**
**Worse prognosis**												
1	GSE4475	Blood cancer	B-cell lymphoma	Berlin (2003-2005)	Hummel	HG-U133A	207777_s_at	158	0.468354	0.00723384	**0.432602**	1,54 [1,12 - 2,11]
**Better prognosis**												
2	GSE5122	Blood cancer	AML	San Diego	Raponi	HG-U133A	207777_s_at	58	0.189655	0.0303372	**-0.355744**	0,70 [0,51 - 0,97]
3	GSE12417-GPL96	Blood cancer	AML	AMLCG (1999-2003)	Metzeler	HG-U133A	207777_s_at	163	0.447853	0.0320862	**-0.589049**	0,55 [0,32 - 0,95]
4	GSE19234	Skin cancer	Melanoma	NYU	Bogunovic	HG-U133_Plus_2	207777_s_at	38	0.105263	0.0042507	**-0.824298**	0,44 [0,25 - 0,77]
5	GSE17536	Colorectal cancer		MCC	Smith	HG-U133_Plus_2	207777_s_at	177	0.220339	0.00149205	**-1.39764**	0,25 [0,10 - 0,59]

SP140L												
**Lp.**	**DATASET**	**CANCER TYPE**	**SUBTYPE**	**COHORT**	**AUTHOR**	**ARRAY TYPE**	**PROBE ID**	**N**	**CUTPOINT**	**COX P-VALUE**	**ln(HR)**	**HR [95% CI low - upp]**
**Worse prognosis**												
1	GSE17537	Colorectal cancer		VMC	Smith	HG-U133_Plus_2	214791_at	55	0.709091	0.0422185	**0.912277**	2,49 [1,03 - 6,00]
2	jacob-00182-MSK	Lung cancer	Adenocarcinoma	MSK	Shedden	HG-U133A	214791_at	104	0.240385	0.0176157	**0.837543**	2,31 [1,16 - 4,61]
**Better prognosis**												
3	GSE5122	Blood cancer	AML	San Diego	Raponi	HG-U133A	214791_at	58	0.465517	0.046767	**-0.463445**	0,63 [0,40 - 0,99]
4	GSE13213	Lung cancer	Adenocarcinoma	Nagoya (1995-1999, 2002-2004)	Tomida	G4112F	A_24_P337012	117	0.247863	0.0121731	**-0.544414**	0,58 [0,38 - 0,89]
5	GSE19234	Skin cancer	Melanoma	NYU	Bogunovic	HG-U133_Plus_2	1565588_at	38	0.157895	0.0309013	**-1.35841**	0,26 [0,07 - 0,88]
**TAF1**												
**Lp.**	**DATASET**	**CANCER TYPE**	**SUBTYPE**	**COHORT**	**AUTHOR**	**ARRAY TYPE**	**PROBE ID**	**N**	**CUTPOINT**	**COX P-VALUE**	**ln(HR)**	**HR [95% CI low - upp]**
**Worse prognosis**												
1	DUKE-OC	Ovarian cancer		Duke	Bild	HG-U133A	216955_at	133	0.68	0.022451	**1.01**	2,75 [1,15 - 6,55]
2	E-TABM-158	Breast cancer		UCSF, CPMC (1989-1997)	Chin	HG-U133A	216711_s_at	117	0.7	0.025502	**0.87**	2,38 [1,11 - 5,10]
3	GSE4475	Blood cancer	B-cell lymphoma	Berlin (2003-2005)	Hummel	HG-U133A	216711_s_at	158	0.88	0.033654	**0.83**	2,28 [1,07 - 4,90]
4	GSE4271-GPL97	Brain cancer	Astrocytoma	MDA	Phillips	HG-U133B	227205_at	77	0.58	0.011783	**0.83**	2,29 [1,20 - 4,35]
**Better prognosis**												
5	jacob-00182-UM	Lung cancer	Adenocarcinoma	UM	Shedden	HG-U133A	216955_at	178	0.15	0.029326	**-0.56**	0,57 [0,35 - 0,95]

6	GSE13213	Lung cancer	Adenocarcinoma	Nagoya (1995-1999, 2002-2004)	Tomida	G4112F	A_32_P192615	117	0.57	0.035635	**-0.67**	0,51 [0,28 - 0,96]
7	GSE13213	Lung cancer	Adenocarcinoma	Nagoya (1995-1999, 2002-2004)	Tomida	G4112F	A_23_P11237	117	0.2	0.010873	**-0.68**	0,51 [0,30 - 0,86]
8	GSE4412-GPL96	Brain cancer	Glioma	UCLA (1996-2003)	Freije	HG-U133A	216711_s_at	74	0.19	0.0061	**-1.36**	0,26 [0,10 - 0,68]
						**TAF1L**						
**Lp.**	**DATASET**	**CANCER TYPE**	**SUBTYPE**	**COHORT**	**AUTHOR**	**ARRAY TYPE**	**PROBE ID**	**N**	**CUTPOINT**	**COX P-VALUE**	**ln(HR)**	**HR [95% CI low - upp]**
**Better prognosis**												
1	GSE17537	Colorectal cancer		VMC	Smith	HG-U133_Plus_2	1553011_at	55	0.18	0.02924	**-2.53**	0,08 [0,01 - 0,77]
2	GSE16581	Brain cancer	Meningioma	UCLA	Lee	HG-U133_Plus_2	1553011_at	67	0.19	0.029159	**-6**	0,00 [0,00 - 0,54]
						**TRIM24**						
**Lp.**	**DATASET**	**CANCER TYPE**	**SUBTYPE**	**COHORT**	**AUTHOR**	**ARRAY TYPE**	**PROBE ID**	**N**	**CUTPOINT**	**COX P-VALUE**	**ln(HR)**	**HR [95% CI low - upp]**
**Worse prognosis**												
1	DUKE-OC	Ovarian cancer		Duke	Bild	HG-U133A	204390_at	133	0.857143	0.0154529	**2.21629**	9,17 [1,53 - 55,15]
2	GSE8970	Blood cancer	AML	San Diego	Raponi	HG-U133A	204391_x_at	34	0.470588	0.00814372	**1.10706**	3,03 [1,33 - 6,87]
3	GSE8970	Blood cancer	AML	San Diego	Raponi	HG-U133A	213301_x_at	34	0.441176	0.0159383	**0.934807**	2,55 [1,19 - 5,45]
4	GSE4271-GPL96	Brain cancer	Astrocytoma	MDA	Phillips	HG-U133A	213301_x_at	77	0.766234	0.00421492	**0.912494**	2,49 [1,33 - 4,65]
5	GSE7390	Breast cancer		Uppsala, Oxford, Stockholm, IGR, GUYT, CRH	Desmedt	HG-U133A	213301_x_at	198	0.565657	0.0183753	**0.629057**	1,88 [1,11 - 3,16]

				(1980-1998)								
6	GSE7390	Breast cancer		Uppsala, Oxford, Stockholm, IGR, GUYT, CRH (1980-1998)	Desmedt	HG-U133A	204391_x_at	198	0.4747 47	0.019614 9	**0.539499**	1,72 [1,09 - 2,70]
7	GSE4271-GPL96	Brain cancer	Astrocytoma	MDA	Phillips	HG-U133A	204391_x_at	77	0.7012 99	0.041587 5	**0.48721**	1,63 [1,02 - 2,60]
8	GSE13507	Bladder cancer		CNUH	Kim	Human-6 v2	ILMN_17112 47	165	0.8121 21	0.002114 73	**0.465281**	1,59 [1,18 - 2,14]
**Better prognosis**												
9	DUKE-OC	Ovarian cancer		Duke	Bild	HG-U133A	213301_x_at	133	0.3082 71	0.007040 9	**-0.445038**	0,64 [0,46 - 0,89]
10	DUKE-OC	Ovarian cancer		Duke	Bild	HG-U133A	204391_x_at	133	0.3984 96	0.011733	**-0.496808**	0,61 [0,41 - 0,90]
11	GSE26712	Ovarian cancer		MSKCC (1990-2003)	Bonome	HG-U133_Plus_2	204390_at	185	0.1243 24	0.010231 4	**-0.673534**	0,51 [0,30 - 0,85]
12	jacob-00182-CANDF	Lung cancer	Adenocarcinoma	CAN/DF	Shedden	HG-U133A	213301_x_at	82	0.1219 51	0.032971 7	**-0.730635**	0,48 [0,25 - 0,94]
**TRIM28**												
**Lp.**	**DATASET**	**CANCER TYPE**	**SUBTYPE**	**COHORT**	**AUTHOR**	**ARRAY TYPE**	**PROBE ID**	**N**	**CUTP OINT**	**COX P-VALUE**	**ln(HR)**	**HR [95% CI low - upp]**
**Worse prognosis**												
1	E-DKFZ-1	Renal cell carcinoma		RZPD	Sueltmann	A-RZPD-20	rzpd,de:hube r1:Reporter:I MAGE:2918 08	59	0.8813 56	0.012556 7	**1.81824**	6,16 [1,48 - 25,69]
2	GSE19234	Skin cancer	Melanoma	NYU	Bogunovic	HG-U133_Plus_2	200990_at	38	0.8947 37	0.028655 1	**1.6158**	5,03 [1,18 - 21,39]
3	GSE31210	Lung cancer	Adenocarcinoma	NCCRI	Okayama	HG-U133_Plus_2	200990_at	204	0.6274 51	0.011705 5	**1.27721**	3,59 [1,33 - 9,68]
4	GSE16560	Prostate cancer		Sweden (1977-1999)	Sboner	6K DASL	DAP4_0261	281	0.5160 14	0.033725 9	**0.53734**	1,71 [1,04 - 2,81]

5	MGH-glioma	Brain cancer	Glioma	CBTTB, MGH, BWH, CH	Nutt	HG-U95A	33425_at	50	0.72	0.0273354	**0.460597**	1,59 [1,05 - 2,39]
**Better prognosis**												
6	GSE9893	Breast cancer		Montpellier, Bordeaux, Turin (1989-2001)	Chanrion	MLRG Human 21K V12,0	11197	155	0.103226	0.00873665	**-0.220106**	0,80 [0,68 - 0,95]
7	GSE8841	Ovarian cancer		Milan (1992-2003)	Marchini	G4100A	10287	81	0.111111	0.0243265	**-1.37057**	0,25 [0,08 - 0,84]
**TRIM33**												
**Lp.**	**DATASET**	**CANCER TYPE**	**SUBTYPE**	**COHORT**	**AUTHOR**	**ARRAY TYPE**	**PROBE ID**	**N**	**CUTPOINT**	**COX P-VALUE**	**ln(HR)**	**HR [95% CI low - upp]**
**Worse prognosis**												
1	GSE19234	Skin cancer	Melanoma	NYU	Bogunovic	HG-U133_Plus_2	212436_at	38	0.736842	0.0361858	**0.844616**	2,33 [1,06 - 5,13]
2	GSE17537	Colorectal cancer		VMC	Smith	HG-U133_Plus_2	210266_s_at	55	0.309091	0.0360051	**0.814598**	2,26 [1,05 - 4,84]
3	GSE9893	Breast cancer		Montpellier, Bordeaux, Turin (1989-2001)	Chanrion	MLRG Human 21K V12,0	16645	155	0.832258	0.00126085	**0.241149**	1,27 [1,10 - 1,47]
**Better prognosis**												
4	GSE31210	Lung cancer	Adenocarcinoma	NCCRI	Okayama	HG-U133_Plus_2	214815_at	204	0.612745	0.0320443	**-0.706547**	0,49 [0,26 - 0,94]
**TRIM66**												
**Lp.**	**DATASET**	**CANCER TYPE**	**SUBTYPE**	**COHORT**	**AUTHOR**	**ARRAY TYPE**	**PROBE ID**	**N**	**CUTPOINT**	**COX P-VALUE**	**ln(HR)**	**HR [95% CI low - upp]**
**Better prognosis**												
1	GSE14764	Ovarian cancer		TOC	Denkert	HG-U133A	213748_at	80	0.1125	0.0377535	**-0.890442**	0,41 [0,18 - 0,95]

2	GSE31210	Lung cancer	Adenocarcinoma	NCCRI	Okayama	HG-U133_Plus_2	213748_at	204	0.279412	0.0291271	**-0.949221**	0,39 [0,16 - 0,91]
						**ZMYND11**						
**Lp.**	**DATASET**	**CANCER TYPE**	**SUBTYPE**	**COHORT**	**AUTHOR**	**ARRAY TYPE**	**PROBE ID**	**N**	**CUTPOINT**	**COX P-VALUE**	**ln(HR)**	**HR [95% CI low - upp]**
**Worse prognosis**												
1	GSE17537	Colorectal cancer		VMC	Smith	HG-U133_Plus_2	1554158_at	55	0.690909	0.024839	**2.68892**	14,72 [1,41 - 154,11]
2	E-TABM-346	Blood cancer	DLBCL	GELA (1998-2000)	Jais	HG-U133A	202136_at	53	0.641509	0.0251919	**1.41143**	4,10 [1,19 - 14,12]
3	GSE14764	Ovarian cancer		TOC	Denkert	HG-U133A	202136_at	80	0.6625	0.0163776	**1.26057**	3,53 [1,26 - 9,87]
4	GSE19234	Skin cancer	Melanoma	NYU	Bogunovic	HG-U133_Plus_2	1554158_at	38	0.447368	0.0470439	**1.04332**	2,84 [1,01 - 7,95]
5	GSE12417-GPL570	Blood cancer	AML	AMLCG (2004)	Metzeler	HG-U133_Plus_2	1554159_a_at	79	0.898734	0.0378686	**0.928119**	2,53 [1,05 - 6,08]
6	GSE19234	Skin cancer	Melanoma	NYU	Bogunovic	HG-U133_Plus_2	1554159_a_at	38	0.447368	0.013549	**0.842393**	2,32 [1,19 - 4,53]
**Better prognosis**												
7	GSE4271-GPL96	Brain cancer	Astrocytoma	MDA	Phillips	HG-U133A	202137_s_at	77	0.766234	0.0139676	**-0.659465**	0,52 [0,31 - 0,87]
8	GSE4412-GPL96	Brain cancer	Glioma	UCLA (1996-2003)	Freije	HG-U133A	202137_s_at	74	0.567568	0.0014541	**-0.681698**	0,51 [0,33 - 0,77]
9	jacob-00182-CANDF	Lung cancer	Adenocarcinoma	CAN/DF	Shedden	HG-U133A	202136_at	82	0.304878	0.0279854	**-0.833771**	0,43 [0,21 - 0,91]
10	GSE4271-GPL96	Brain cancer	Astrocytoma	MDA	Phillips	HG-U133A	202136_at	77	0.571429	0.00128598	**-0.914117**	0,40 [0,23 - 0,70]
11	GSE1456-GPL96	Breast cancer		Stockholm (1994-1996)	Pawitan	HG-U133A	202136_at	159	0.169811	0.0281407	**-0.918207**	0,40 [0,18 - 0,91]
12	GSE4412-GPL96	Brain cancer	Glioma	UCLA (1996-2003)	Freije	HG-U133A	202136_at	74	0.608108	0.000807635	**-0.960253**	0,38 [0,22 - 0,67]
13	GSE11117	Lung cancer	NSCLC	Basel (2002-2005)	Baty	Novachip human 34,5k	H300019781	41	0.170732	0.00394934	**-0.983298**	0,37 [0,19 - 0,73]

14	GSE31210	Lung cancer	Adenocarcinoma	NCCRI	Okayama	HG-U133_Plus_2	202137_s_at	204	0.367647	0.00947246	**-1.37738**	0,25 [0,09 - 0,71]
15	MGH-glioma	Brain cancer	Glioma	CBTTB, MGH, BWH, CH	Nutt	HG-U95A	33864_at	50	0.16	0.00760017	**-1.56319**	0,21 [0,07 - 0,66]
16	GSE31210	Lung cancer	Adenocarcinoma	NCCRI	Okayama	HG-U133_Plus_2	202136_at	204	0.348039	0.0294937	**-1.81595**	0,16 [0,03 - 0,83]
						**ZMYND8**						
**Lp.**	**DATASET**	**CANCER TYPE**	**SUBTYPE**	**COHORT**	**AUTHOR**	**ARRAY TYPE**	**PROBE ID**	**N**	**CUTPOINT**	**COX P-VALUE**	**ln(HR)**	**HR [95% CI low - upp]**
**Worse prognosis**												
1	GSE11117	Lung cancer	NSCLC	Basel (2002-2005)	Baty	Novachip human 34,5k	H300021910	41	0.804878	0.0102694	**1.06627**	2,90 [1,29 - 6,56]
2	HARVARD-LC	Lung cancer	Adenocarcinoma	Harvard	Beer	HG-U95A	36957_at	84	0.238095	0.00495721	**0.963488**	2,62 [1,34 - 5,13]
3	GSE4475	Blood cancer	B-cell lymphoma	Berlin (2003-2005)	Hummel	HG-U133A	209048_s_at	158	0.481013	0.0217145	**0.74768**	2,11 [1,12 - 4,00]
4	HARVARD-LC	Lung cancer	Adenocarcinoma	Harvard	Beer	HG-U95A	842_at	84	0.821429	0.025951	**0.57151**	1,77 [1,07 - 2,93]
5	GSE7390	Breast cancer		Uppsala, Oxford, Stockholm, IGR, GUYT, CRH (1980-1998)	Desmedt	HG-U133A	209048_s_at	198	0.29798	0.042365	**0.433367**	1,54 [1,02 - 2,34]
6	GSE16560	Prostate cancer		Sweden (1977-1999)	Sboner	6K DASL	DAP2_3392	281	0.120996	0.00336388	**0.310978**	1,36 [1,11 - 1,68]
**Better prognosis**												
7	GSE31210	Lung cancer	Adenocarcinoma	NCCRI	Okayama	HG-U133_Plus_2	230533_at	204	0.72549	0.0151036	**-1.4782**	0,23 [0,07 - 0,75]
8	GSE31210	Lung cancer	Adenocarcinoma	NCCRI	Okayama	HG-U133_Plus_2	214795_at	204	0.210784	5.47904e-8	**-1.96791**	0,14 [0,07 - 0,28]
9	GSE17537	Colorectal cancer		VMC	Smith	HG-U133_Plus_2	230533_at	55	0.163636	0.0102895	**-3.44268**	0,03 [0,00 - 0,44]

**DATASET**	Dataset ID from the GEO database
**CANCER TYPE**	Cancer type
**SUBTYPE**	Subtype of cancer
**COHORT**	The name of patients' cohort
**AUTHOR**	The name of the contributor
**ARRAY TYPE**	Type of array used in the study
**PROBE ID**	Identification number of probe in the array
**N**	Number of patients
**CUTPOINT**	Optimal cutpoint for the overall survival analysis
**COX P-VALUE**	The statistical significance for Cox proportional hazard ratio model
**ln(HR)**	ln-normalized hazard ratio
**HR [95% CI low - upp]**	Lower and upper confidence interval for HR

Supplementary Table 4
Additional GEO datasets from the R2 database.

Abbreviation	Tumor Type	Platform	Normalization	Number of samples	Author	GEO accesion number	Ref.
Bladder	Tumor Bladder	ilmnht12v4	custom	142	Choi	GSE48075	4
Breast	Tumor Breast	u133p2	MAS5.0	683	Yu	GSE102484	5
Cervix	Tumor Cervix	ilmnht12v4	custom	300	Kim	GSE44001	6
Glioma	Tumor Glioma	u133p2	MAS5.0	284	French	GSE16011	7
Kidney	Tumor Kidney	u133p2	MAS5.0	261	EXPO	GSE2109	
Liver	Tumor Liver (HCC-CC)	u133a	MAS5.0	90	Kim	GSE15765	8
Lung	Lung Adenocarcinoma	u133a	MAS5.0	107	Jen	GSE10072	9
Melanoma	Tumor Melanoma	ilmnht12v4	custom	214	Jonsson	GSE65904	10
Pancreas	Pancreatic ductal adenocarcinoma (ICGC)	ilmnht12v4	complex	91	Perez	GSE36924	11
Sarcoma	Tumor Sarcoma	u133a	MAS5.0	137	Filion		12

Supplementary Table 5
The statistical significance corresponding to Figure 2.

	mRNA-SI	KAT2B	KAT2A	EP300	TAF1
**Kruskal-Wallis test (p-val)**	**1,00E-15**	**1,85E-07**	**4,00E-15**	**0,00278325**	**1,25E-09**
**Dunn's Multiple Comparisons test (adj. p-val):**					
**Basal vs HER2+**	**0,048372944**	**0,01802352**	**1,73E-10**	1	1
**Basal vs Lum A**	**1,00E-15**	**0,00298472**	0,45875353	**0,02548589**	**3,64E-08**
**Basal vs Lum B**	0,250890155	0,1655623	1	**0,03895552**	**0,00057688**
**Basal vs Normal**	**1,00E-15**	**3,17E-08**	**3,62E-05**	**0,0020375**	**0,00010535**
**HER2+ vs Lum A**	**1,00E-15**	1	**1,06E-08**	1	**0,00042013**
**HER2+ vs Lum B**	1	1	**8,64E-12**	1	**0,02560925**
**HER2+ vs Normal**	**1,62E-07**	0,96054073	**0,04572528**	0,66935227	**0,00613869**
**Lum A vs Lum B**	**1,00E-15**	1	0,09437831	1	1
**Lum A vs Normal**	0,114302561	**0,00193727**	**0,0023355**	0,99179123	1
**Lum B vs Normal**	**1,00E-15**	**0,00142735**	**2,84E-06**	1	1
	**TAF1L**	**BRPF1**	**BRD1**	**BRPF3**	**BRD8**
**Kruskal-Wallis test (p-val)**	**6,64E-07**	**5,70E-12**	**0,00015952**	**2,80E-05**	**1,00E-15**
**Dunn's Multiple Comparisons test (adj. p-val):**					
**Basal vs HER2+**	1	**6,65E-07**	1	**0,04664192**	1
**Basal vs Lum A**	**8,42E-06**	**5,83E-12**	**0,00866706**	**2,63E-05**	**1,00E-15**
**Basal vs Lum B**	**0,00228302**	**1,83E-07**	**0,00015938**	**7,38E-05**	**1,00E-15**
**Basal vs Normal**	**0,00016295**	**5,60E-06**	0,61700837	**0,0082631**	**4,28E-05**
**HER2+ vs Lum A**	**0,02069214**	1	0,60351968	1	**4,40E-14**
**HER2+ vs Lum B**	0,13915219	1	**0,0410737**	1	**2,44E-13**
**HER2+ vs Normal**	**0,02174492**	1	1	1	0,05957796
**Lum A vs Lum B**	1	1	0,59744438	1	1
**Lum A vs Normal**	1	1	1	1	**1,20E-08**
**Lum B vs Normal**	1	1	0,31640569	1	**5,58E-08**
	**ASH1L**	**KMT2A**	**SMARCA2**	**SMARCA4**	**PBRM1**
**Kruskal-Wallis test (p-val)**	**3,63E-09**	**4,94E-06**	**1,00E-15**	**1,00E-15**	**3,28E-05**
**Dunn's Multiple Comparisons test (adj. p-val):**					
**Basal vs HER2+**	1	1	1	0,66836823	1
**Basal vs Lum A**	**6,28E-07**	0,20309526	**5,14E-06**	**1,00E-15**	**0,00042675**
**Basal vs Lum B**	**0,02051282**	1	0,95380912	**2,03E-09**	**0,00287054**
**Basal vs Normal**	**0,00133926**	**0,00050132**	**0,00075226**	**1,61E-11**	**0,00026056**
**HER2+ vs Lum A**	**3,19E-05**	0,2772174	**1,84E-06**	**1,08E-05**	0,397094
**HER2+ vs Lum B**	**0,01786268**	1	1	**0,02722338**	0,43624164
**HER2+ vs Normal**	**0,00179207**	**0,00223124**	**4,74E-05**	**0,00139222**	0,09185796
**Lum A vs Lum B**	0,70945143	**0,02817925**	**3,24E-12**	0,19699508	1
**Lum A vs Normal**	1	0,07040635	1	1	1
**Lum B vs Normal**	1	**4,55E-05**	**1,92E-07**	1	1

	BRD7	BRD9	BAZ1A	BAZ1B	BAZ2A
**Kruskal-Wallis test (p-val)**	**1,00E-15**	**5,04E-09**	**1,00E-15**	**1,00E-15**	**1,00E-15**
**Dunn's Multiple Comparisons test (adj. p-val):**					
**Basal vs HER2+**	1	**3,13E-07**	0,0588999	1	**1,08E-06**
**Basal vs Lum A**	**1,00E-15**	**0,00600805**	**6,97E-10**	**2,65E-13**	**1,00E-15**
**Basal vs Lum B**	**0,02591571**	**0,00010315**	1	1	**1,00E-15**
**Basal vs Normal**	**0,00211293**	**5,72E-06**	1	**0,0002493**	**1,84E-10**
**HER2+ vs Lum A**	**1,18E-12**	**0,00171437**	**1,73E-13**	**0,00384079**	0,07917836
**HER2+ vs Lum B**	0,09310078	0,24524948	**0,04556999**	1	**0,02777332**
**HER2+ vs Normal**	**0,01326317**	1	**0,00311609**	0,78685685	1
**Lum A vs Lum B**	**1,75E-10**	0,57979347	**1,93E-10**	**7,15E-10**	1
**Lum A vs Normal**	**1,34E-05**	0,05115759	**5,00E-05**	0,47819658	**0,01400126**
**Lum B vs Normal**	1	1	1	**0,01150069**	**0,0055233**

	BAZ2B	BPTF	CECR2	ATAD2	ATAD2B
**Kruskal-Wallis test (p-val)**	**1,00E-15**	**1,22E-11**	**5,81E-10**	**1,00E-15**	**1,32E-09**
**Dunn's Multiple Comparisons test (adj. p-val):**					
**Basal vs HER2+**	0,09776086	0,34010029	**8,68E-07**	**0,00018377**	1
**Basal vs Lum A**	**1,00E-15**	**0,01407238**	0,594337621	**1,00E-15**	**8,34E-07**
**Basal vs Lum B**	**2,06E-07**	**2,59E-12**	**3,04E-06**	1	1
**Basal vs Normal**	**1,81E-13**	**0,01056379**	**0,03208332**	**1,00E-14**	1
**HER2+ vs Lum A**	**9,77E-05**	1	**3,41E-05**	**4,04E-06**	**4,46E-05**
**HER2+ vs Lum B**	0,96893968	**0,0059158**	1	**0,00012738**	0,57217472
**HER2+ vs Normal**	**0,00374107**	1	**0,045265548**	0,27530289	0,65229414
**Lum A vs Lum B**	**0,00128071**	**9,49E-08**	**0,000117059**	**1,00E-15**	**0,00229773**
**Lum A vs Normal**	1	1	0,757013006	**0,01022061**	**0,01357767**
**Lum B vs Normal**	0,10348672	**0,00412864**	0,71282551	**2,00E-15**	1

	BRD3	BRD4	BRD2	BRDT	TRIM24
**Kruskal-Wallis test (p-val)**	**1,00E-15**	**1,35E-10**	**0,00026729**	**1,00E-15**	**3,85E-12**
**Dunn's Multiple Comparisons test (adj. p-val):**					
**Basal vs HER2+**	**2,01E-09**	**0,00036457**	1	**0,00357501**	1
**Basal vs Lum A**	**1,00E-15**	**2,47E-10**	1	**1,00E-15**	**6,75E-06**
**Basal vs Lum B**	**8,45E-12**	**5,73E-09**	**0,04535801**	**1,00E-15**	1
**Basal vs Normal**	**2,58E-05**	**0,00093495**	1	**1,21E-11**	0,25930996
**HER2+ vs Lum A**	1	1	0,31652583	**4,97E-05**	**2,76E-05**
**HER2+ vs Lum B**	1	1	**0,00923071**	**2,49E-05**	1
**HER2+ vs Normal**	0,15233819	1	1	0,30443435	0,05353225
**Lum A vs Lum B**	1	1	0,2765322	1	**1,28E-08**
**Lum A vs Normal**	0,07624544	1	0,09380604	0,07124963	0,55580958
**Lum B vs Normal**	0,54686658	0,66517718	**0,0009417**	**0,02733194**	**0,03011082**

	TRIM28	TRIM33	TRIM66	SP100	SP110
**Kruskal-Wallis test (p-val)**	**0,00057785**	0,06012135	**1,00E-15**	**1,00E-15**	**5,17E-13**

**Dunn's Multiple Comparisons test (adj. p-val):**					
**Basal vs HER2+**	1	1	1	1	**7,46E-10**
**Basal vs Lum A**	1	1	**1,00E-15**	0,31218045	**5,99E-08**
**Basal vs Lum B**	1	1	**1,00E-15**	**1,00E-15**	**1,87E-10**
**Basal vs Normal**	**0,00101051**	1	**3,40E-12**	0,11865757	**2,96E-05**
**HER2+ vs Lum A**	1	1	**1,00E-15**	1	**0,01542354**
**HER2+ vs Lum B**	1	0,49508576	**8,38E-09**	**6,60E-14**	1
**HER2+ vs Normal**	**0,02339082**	0,70664827	**1,38E-05**	0,80998876	0,09484272
**Lum A vs Lum B**	1	0,20887726	**0,00061173**	**1,00E-15**	0,23160132
**Lum A vs Normal**	**0,00830188**	0,50916928	**4,13E-06**	1	1
**Lum B vs Normal**	**0,00179477**	1	1	**1,00E-15**	1

	**SP140**	**SP140L**	**ZMYND8**	**ZMYND11**	**BRWD1**
**Kruskal-Wallis test (p-val)**	**1,94E-10**	0,42456235	**1,00E-15**	**1,46E-09**	**1,00E-15**
**Dunn's Multiple Comparisons test (adj. p-val):**					
**Basal vs HER2+**	1	1	**1,00E-15**	0,16001354	**0,00745205**
**Basal vs Lum A**	**3,25E-07**	1	**1,00E-15**	**0,0001023**	**1,00E-15**
**Basal vs Lum B**	**0,00388464**	1	**1,00E-15**	**0,04033719**	**1,00E-15**
**Basal vs Normal**	0,1179481	1	**2,40E-14**	**0,03816562**	**1,39E-06**
**HER2+ vs Lum A**	**7,19E-07**	1	0,3381893	**6,71E-08**	0,22591154
**HER2+ vs Lum B**	**0,00047793**	1	1	**3,45E-05**	**0,01065941**
**HER2+ vs Normal**	**0,01046479**	1	0,0599602	**4,04E-05**	1
**Lum A vs Lum B**	1	1	**1,59E-05**	1	0,52060103
**Lum A vs Normal**	0,37374755	1	1	1	1
**Lum B vs Normal**	1	1	**1,38E-05**	1	**0,04239716**

	**BRWD3**	**PHIP**
**Kruskal-Wallis test (p-val)**	**0,00060041**	0,30506524
**Dunn's Multiple Comparisons test (adj. p-val):**		
**Basal vs HER2+**	1	1
**Basal vs Lum A**	1	1
**Basal vs Lum B**	**0,04803785**	1
**Basal vs Normal**	0,44628739	1
**HER2+ vs Lum A**	0,74094748	1
**HER2+ vs Lum B**	1	0,71993182
**HER2+ vs Normal**	1	0,61535073
**Lum A vs Lum B**	**0,00164822**	1
**Lum A vs Normal**	0,08436417	1
**Lum B vs Normal**	1	1

Supplementary Figure 1.

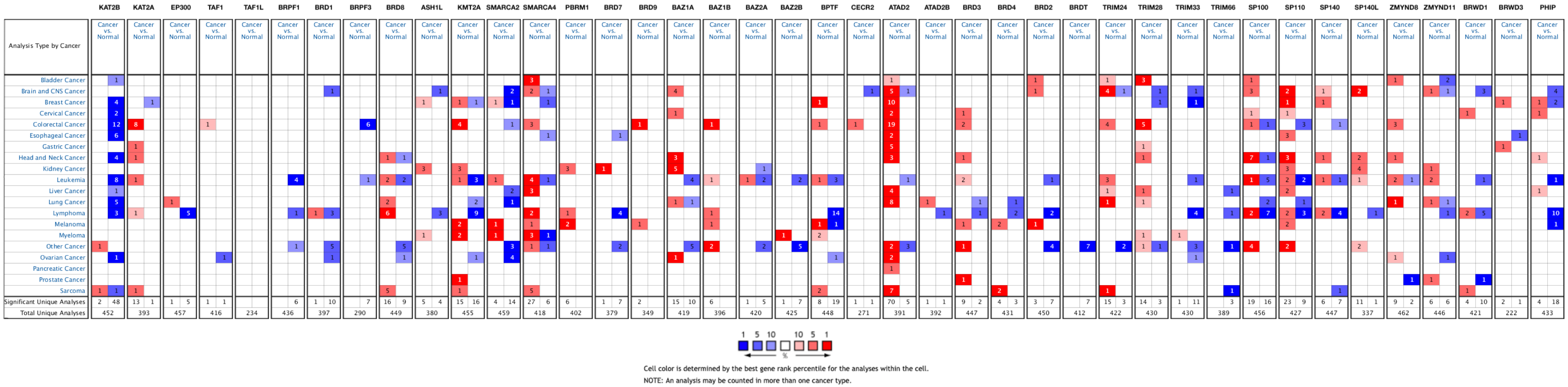

Supplementary Figure 1. Transcription levels of BrD family members in different types of cancers using the Oncomine database. The graphic was generated using Oncomine, indicating the numbers of datasets with statistically significant ($p < 0.01$) over-expression (red) or under-expression (blue) of BrD members' mRNA (cancer vs. corresponding normal tissue). The threshold was designed with the following parameters: p-value = 0.01, fold change = 2, and gene ranking = 10%. The numbers in the boxes represent the number of analyses that met these thresholds.

Supplementary Figure 2.

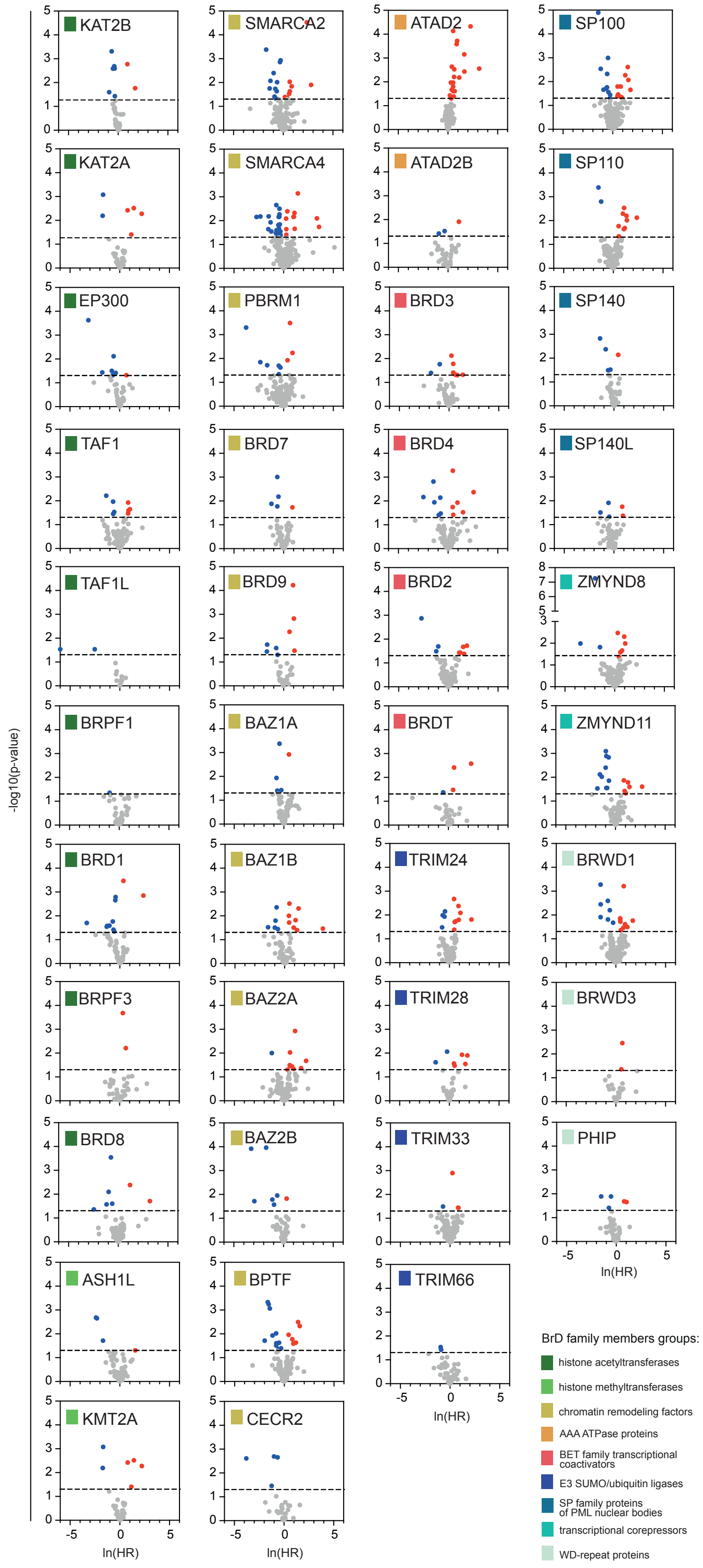

Supplementary Figure 2. The association of BrD family members' expression with cancer patients' overall survival using the Prognoscan database. Dot plots demonstrate the ln-normalized hazard ratio of death - either lower (blue dots) or higher (red dots) - for cancer patients expressing higher levels of specific BrD members. Only statistically significant data ($p < 0.05$) are color-coded (for further details see Supplementary Table 3).

Supplementary Figure 3A.

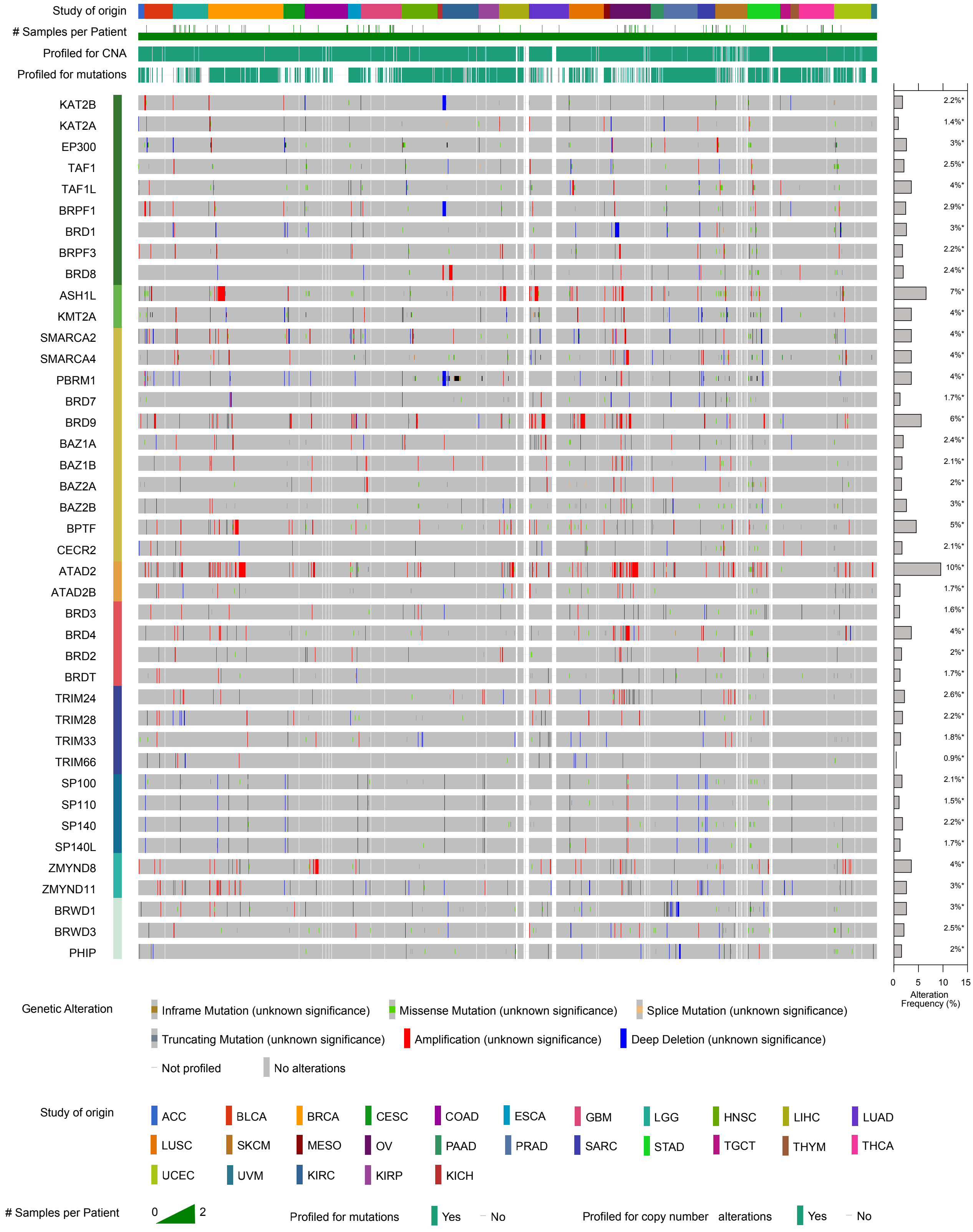

Supplementary Figure 3B.

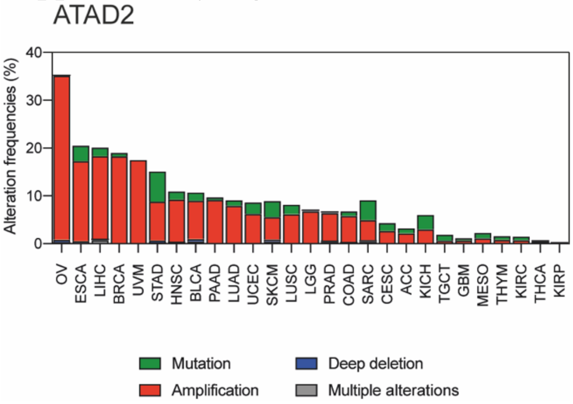

Supplementary Figure 3. The alteration frequencies in BrD family members according to cBioportal database. (A) Distinct patterns of mutations are observed in BrD members across solid tumors. TCGA tumors are color-coded. The types of genetic alterations (inframe, missense, splice, truncating mutation, amplification, or deep deletion) are described in the legend. (B) The frequencies of alterations in *ATAD2* gene across 27 solid TCGA tumor types. Green – Inframe, missense, splice, or truncating mutations; Red – amplifications; Blue – deep deletions; Grey – multiple alterations. Data are shown as the proportion of profiled samples in each tumor type.

Supplementary Figure 4.

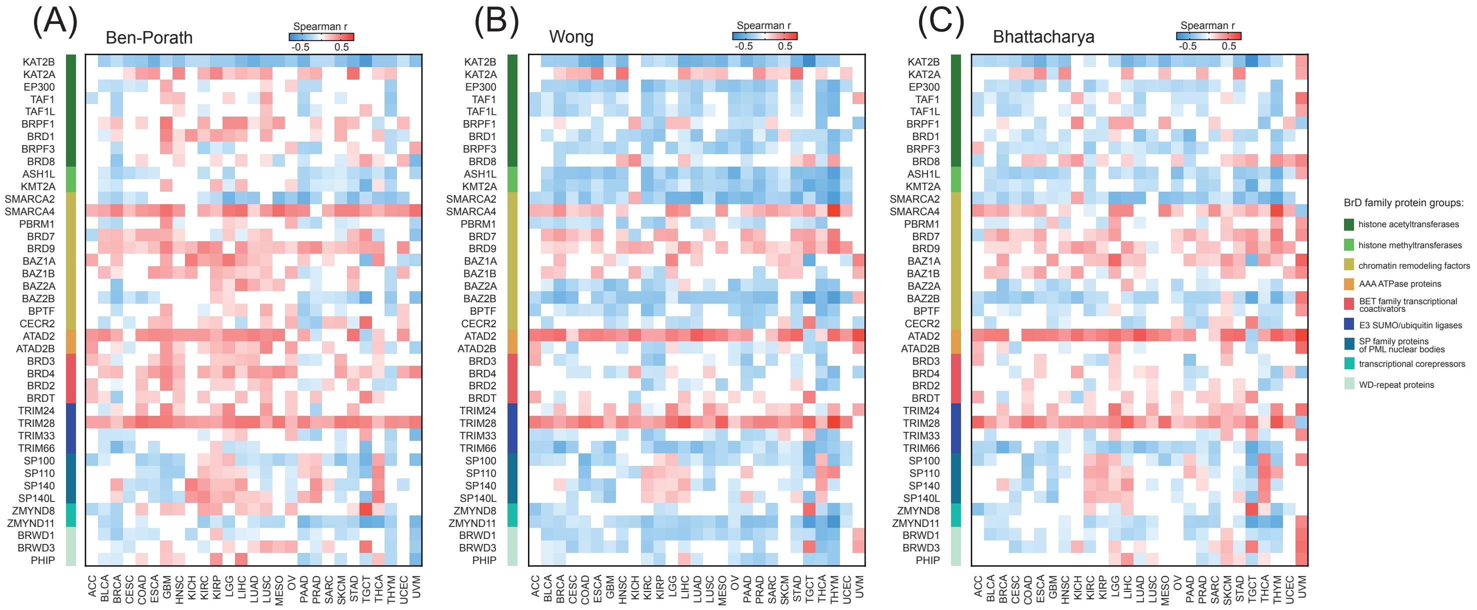

Supplementary Figure 4. The association between BrD family members' expression and cancer stemness (based on Ben-Porath, Wong, Bhattacharya). (A-C) The heatmap of Spearman's correlation between BrD family members' expression and distinct stemness scores: (A) Ben-Porath ES core signature, (B) Wong ESC core signature, and (C) Bhattacharya ESC signature.

Supplementary Figure 5.

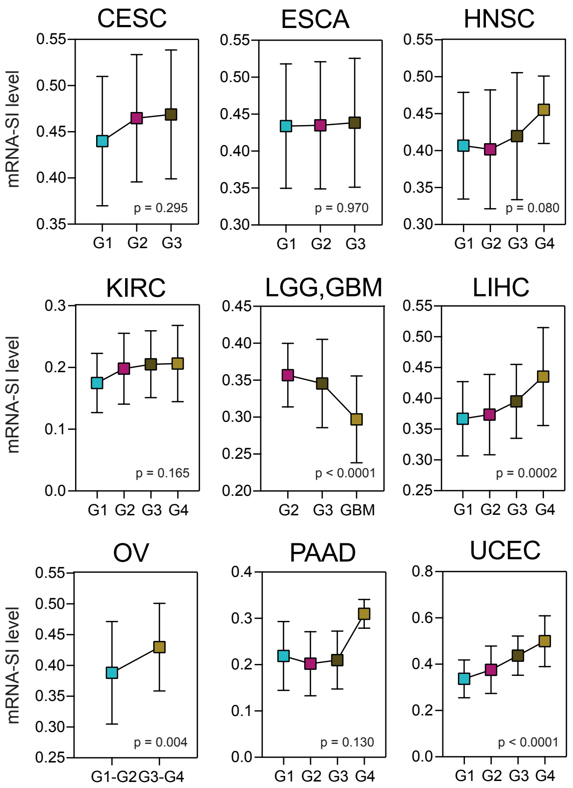

Supplementary Figure 5. Higher grade tumors clearly exhibit stemness characteristics mirrored by higher values of the mRNA-SI score.

Supplementary Figure 6.

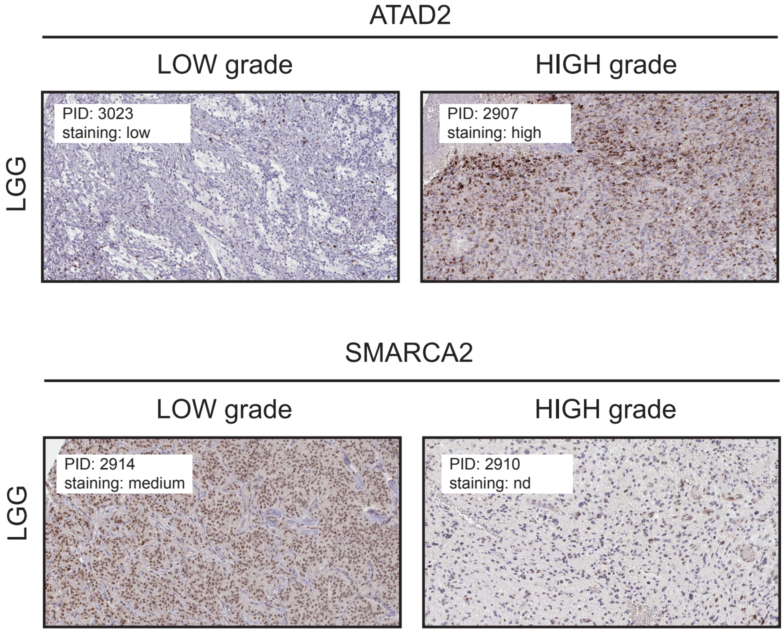

Supplementary Figure 6. The level of ATAD2 and SMARCA2 proteins in lower and higher grade LGG tumors as determined with immunohistochemistry staining with CAB056158 and CAB037276 antibodies, respectively (from The Human Protein Atlas database, https://www.proteinatlas.org/). PID - patient ID; staining - the antibody staining in the annotated cell types in the current human tissue is reported as not detected, low, medium, or high. This score is based on the staining intensity and fraction of stained cells.

Supplementary Figure 7.

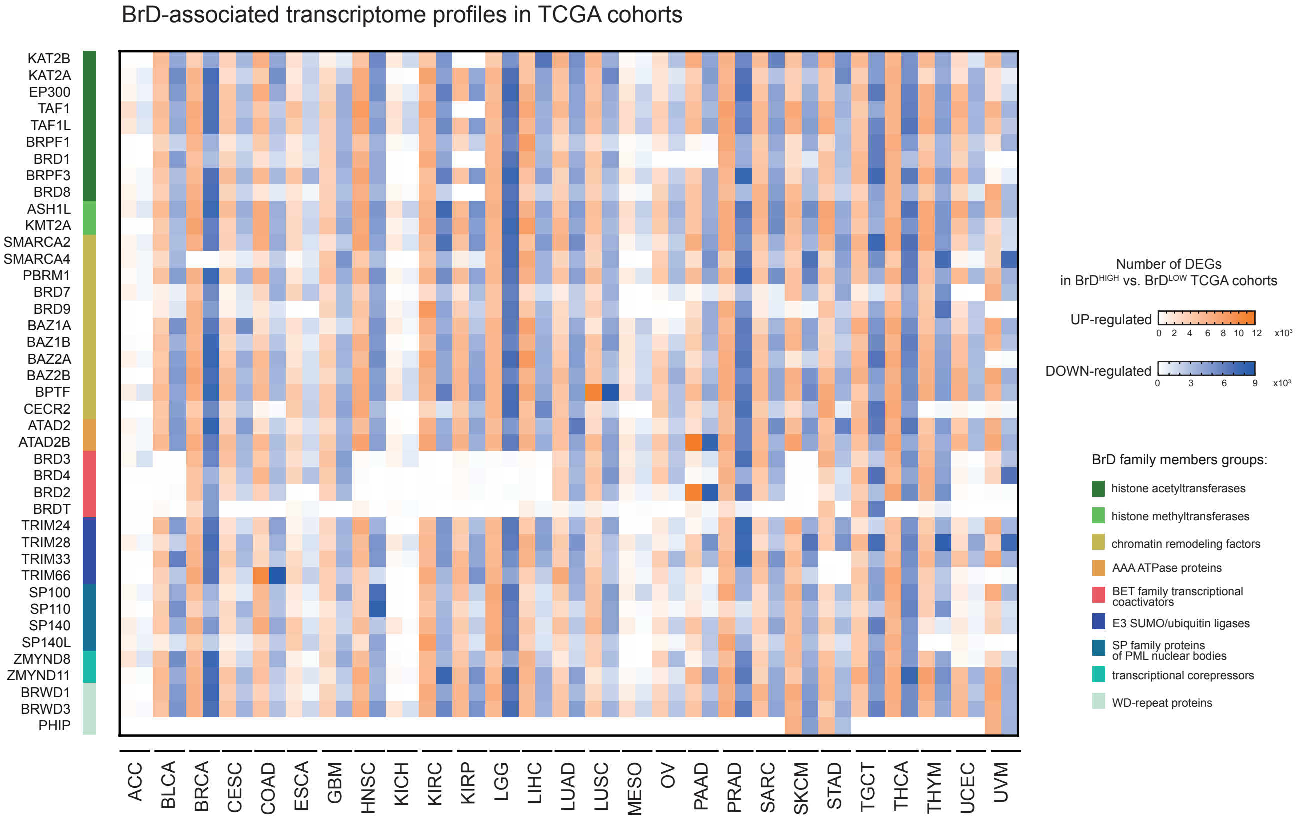

Supplementary Figure 7. The transcriptome profiles associated with BrD family members' expression in solid tumors. The number of DEGs, either upregulated (orange) or downregulated (blue), in BrD^{HIGH} vs. BrD^{LOW}-expressing TCGA cohorts (using the mean expression of BrD protein as a cut-off).

Supplementary Figure 8.

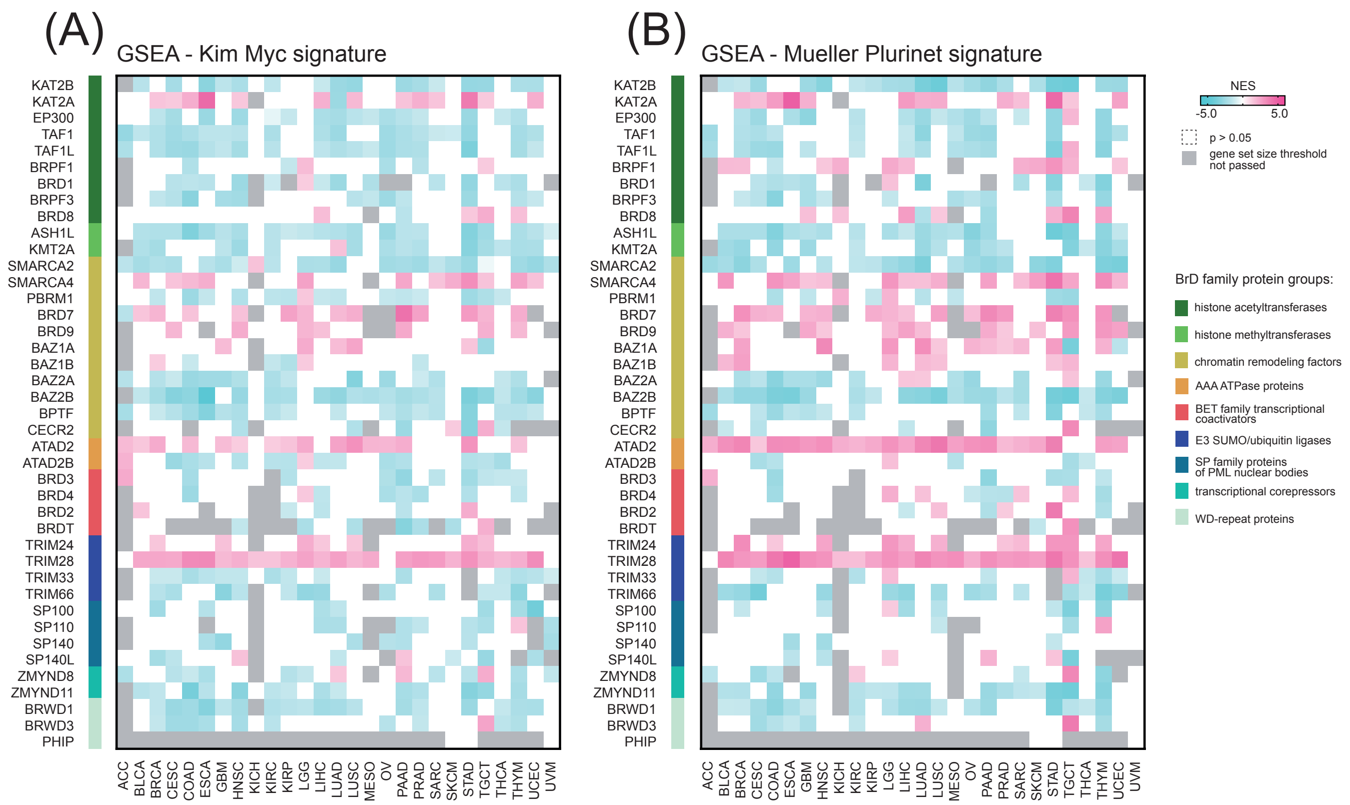

Supplementary Figure 8. The GSEA analyses of BrD-associated gene signatures using previously defined stemness signatures as references. (A,B) The Gene Set Enrichment Analysis (GSEA) using significantly differentially expressed genes (DEGs, $p < 0.05$, FDR < 0.05) in TCGA patients divided into low- or high-expressing BrD cohorts (using the mean expression of each BrD family member as a cut-off) was performed with (A) the Kim Myc (13) or (B) the Mueller Plurinet signature (14) as a reference. The heatmaps present the normalized enrichment score (NES). White - no statistical significance ($p > 0.05$); grey - the gene set size thresholds were not reached or no DEGs were detected.

Supplementary Figure 9.

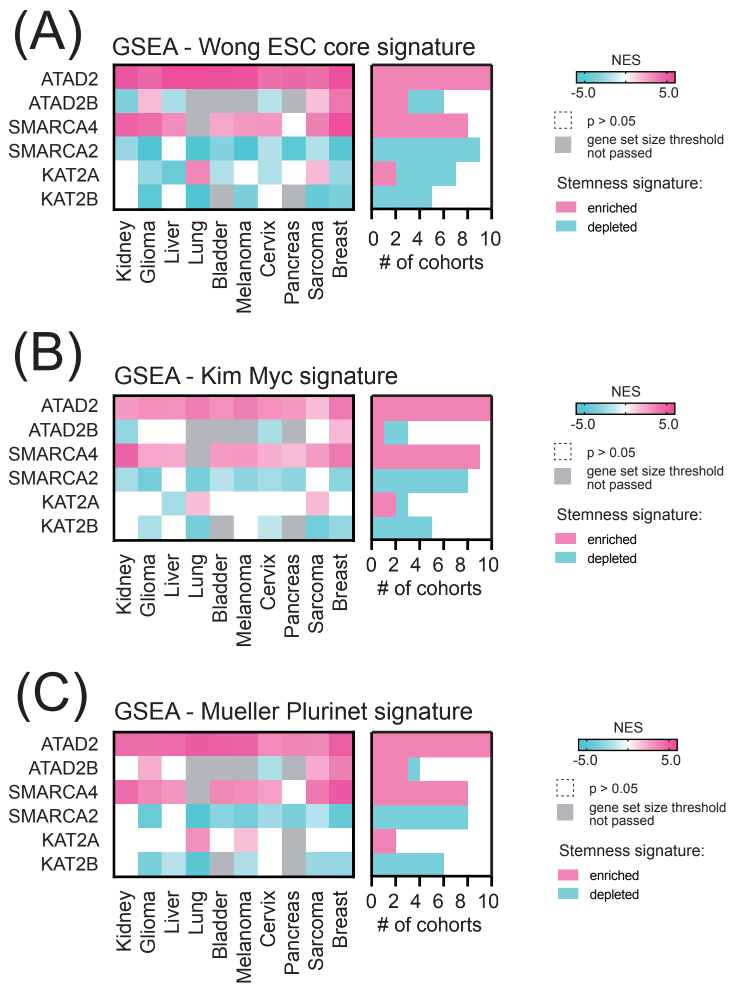

Supplementary Figure 9. The GSEA analyses of BrD-associated gene signatures from additional GEO datasets using previously defined stemness signatures as references. (A-C) The Gene Set Enrichment Analysis (GSEA) using significantly coexpressed genes (Spearman correlation, $p < 0.05$, $FDR < 0.01$) to selected BrD family members in additional GEO datasets (for further details see Supplementary Table 4, page 39) performed with (A) the Wong ESC core, (B) the Kim Myc (13) or (C) the Mueller Plurinet (14) signature as a reference, respectively. The heatmaps present the normalized enrichment score (NES). White - no statistical significance ($p > 0.05$); grey - the gene set size thresholds were not reached or no DEGs were detected.

Supplementary Figure 10.

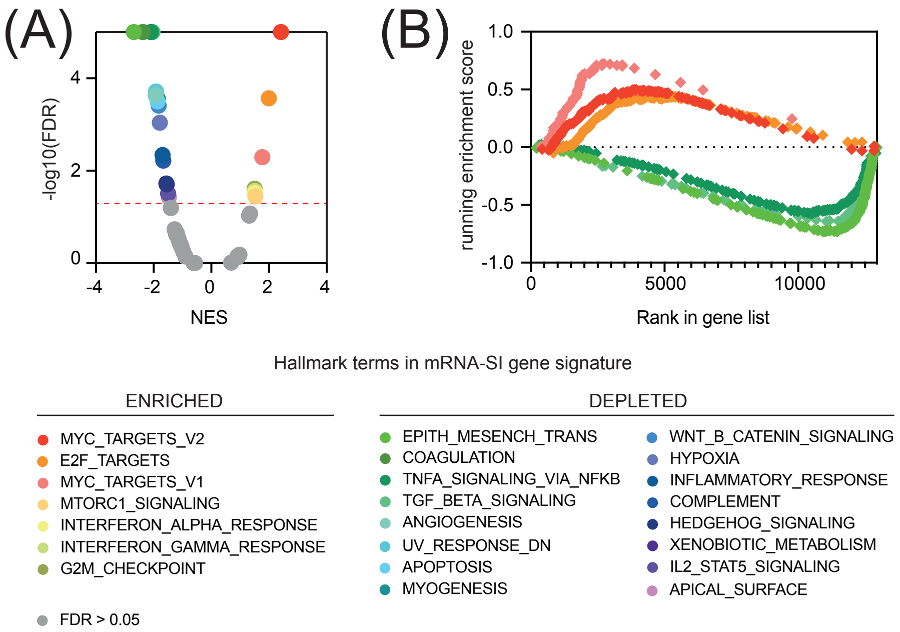

Supplementary Figure 10. The GSEA analysis of mRNA-SI gene signature using MSigDB Hallmarks (v7.4) gene sets as a reference. (A) Dot plot of Hallmark terms enriched/depleted in mRNA-SI gene signature. All significantly enriched or depleted Hallmark terms are color-coded. Grey dots - Hallmark terms with FDR > 0.05; NES - normalized enrichment score. (B) The enrichment plots for 3 top enriched (MYC_targets_V1, MYC_targets_V2, E2F_targets) and 3 top depleted (Epithelial_mesenchymal_transition, TNFa_signaling_via_NFkB, TGF_beta_signaling) Hallmark terms (color-coded as presented in the legend below).

Supplementary Figure 11.

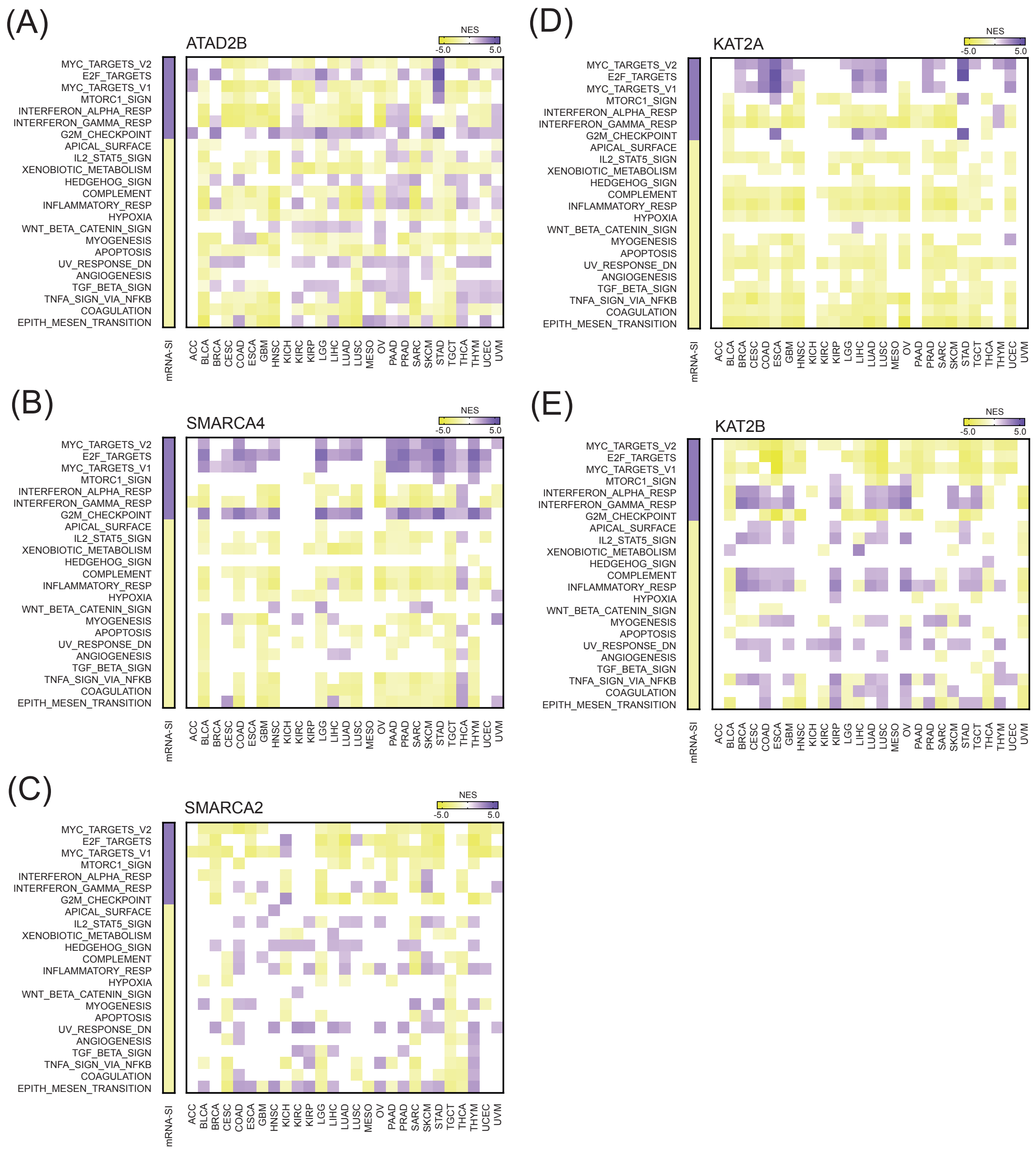

Supplementary Figure 11. The GSEA analysis of selected BrD-associated transcriptome profiles in solid tumors with MSigDB Hallmark (v7.4) collection as a reference. (A-E). The GSEA using significantly differentially expressed genes (DEGs, $p < 0.05$, $FDR < 0.05$) in TCGA patients divided into low- or high-expressing *ATAD2B* (A), *SMARCA4* (B), *SMARCA2* (C), *KAT2A* (D) and *KAT2B* (E) cohorts (using the mean expression of each gene as a cut-off) was performed with the MSigDB Hallmark (v7.4) collection as a reference. The heatmap presents the normalized enrichment score (NES). White - no statistical significance ($p > 0.05$) or no DEGs were detected. Only those Hallmark termes, that were previously determined as significantly enriched (violet) or depleted (yellow) in the mRNA-SI gene signature are presented in the heatmap.

Supplementary Figure 12.

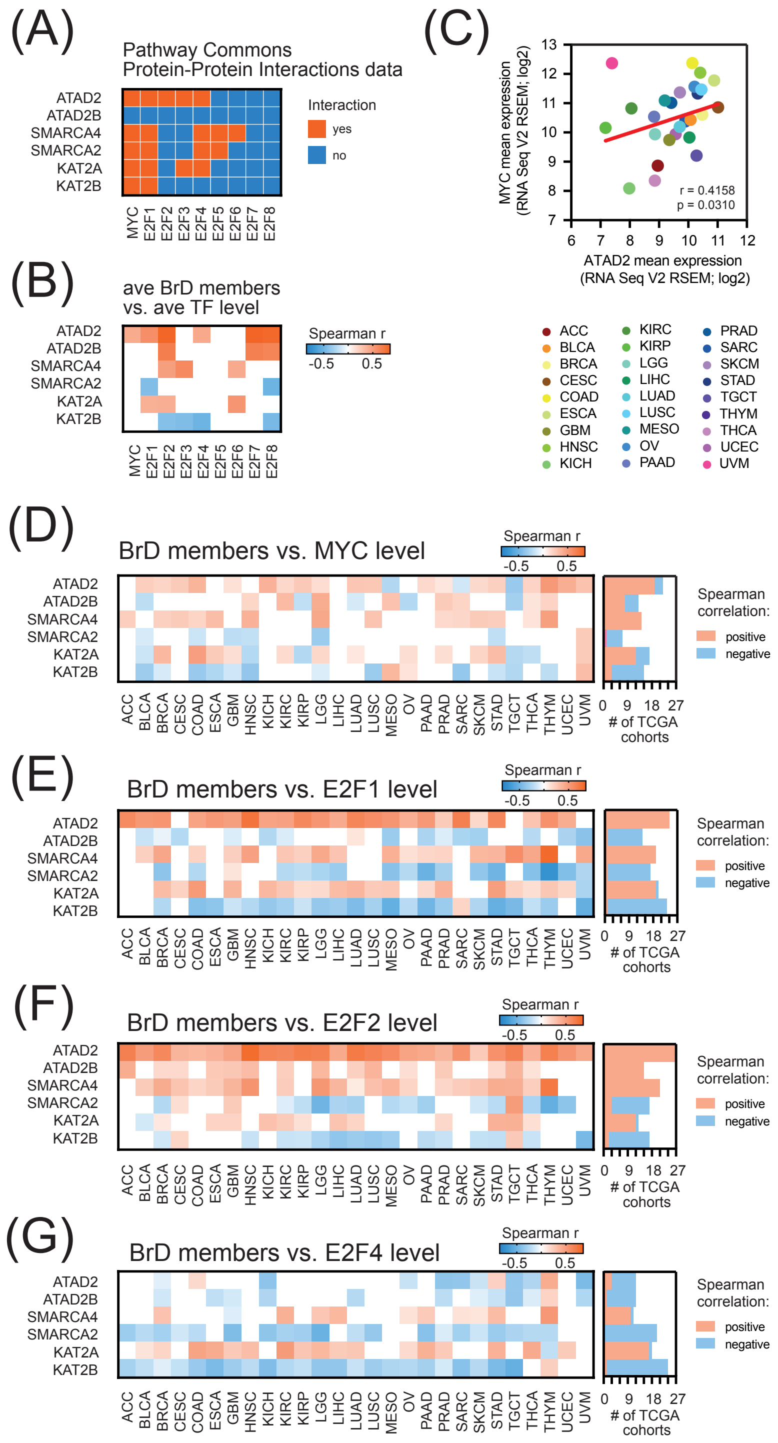

Supplementary Figure 12. The association between selected BrD members and c-Myc and E2F family transcription factors. (A) The physical interaction between selected BrD members and c-Myc and E2F transcription factors according to the Pathway Commons Protein-Protein Interactions dataset. (B) The association between the cohort mean *MYC* and E2F family members' level and the cohort mean expression (log2-normalized) of BrD genes across 27 TCGA cohorts. (C) Dot plots of Spearman correlation between the cohort mean expression of *ATAD2* and the cohort mean *MYC* level. (D-G). The heatmaps of Spearman's correlation between selected BrD members gene expression and (D) *MYC*, (E) *E2F1*, (F) *E2F2*, and (G) *E2F4* transcription factors expression across 27 TCGA tumor types. The number of TCGA cohorts characterized with either a positive (orange) or negative (blue) correlation between the expression of selected markers are shown.

Supplementary Figure 13.

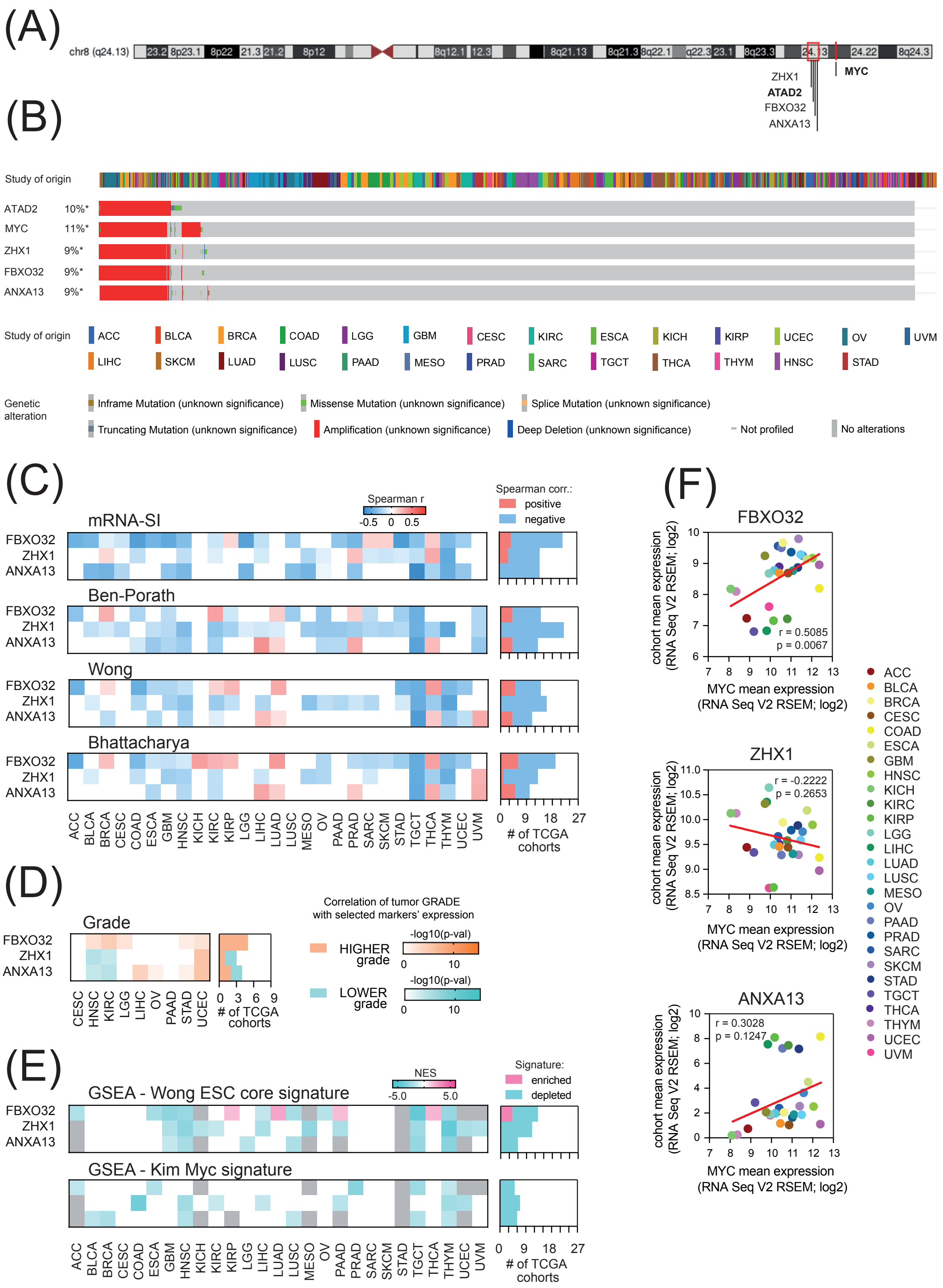

Supplementary Figure 13. Validation with additional genes encoded within the long arm of chromosome 8 (ATAD2 neighborhood) confirmed the specificity of *ATAD2* and *MYC* as well as *ATAD2* and cancer stemness association. (A) *ZHX1*, *FBXO32*, and *ANXA13* are close neighbors of *ATAD2* gene, encoded within the same region of chromosome 8. (B) *ATAD2, MYC*, *ZHX1*, *FBXO32*, and *ANXA13* are together amplified in TCGA tumors. (C) The heatmaps of Spearman's correlation between *ZHX1*, *FBXO32*, and *ANXA13* gene expression and mRNA stemness indices (mRNA-SI, Ben-Porath, Wong, Bhattacharya) across 27 TCGA tumor types.

The number of TCGA cohorts characterized with either a positive (red) or negative (blue) correlation between the expression of selected markers and specific stemness index are shown. (D) The association between selected markers encoded within chromosome 8 q24.13 and tumor grade - either lower (blue) or higher grade (orange), as determined with Spearman correlation test (-log10(p-value)). (E) The Gene Set Enrichment Analysis (GSEA) using significantly differentially expressed genes (DEGs, $p < 0.05$, FDR < 0.05) in TCGA patients divided into low- or high-expressing markers from chromosome 8 q24.13 (*ZHX1*, *FBXO32*, and *ANXA13*) was performed with the stemness signature (Wong_ESC_Core - top panel, Kim_Myc - bottom panel) as a reference. The heatmaps present the normalized enrichment score (NES). White - no statistical significance ($p > 0.05$); grey - the gene set size thresholds were not reached or no DEGs were detected. (F) Dot plots of Spearman correlation between the cohort mean expression of selected markers encoded within the long arm of chromosome 8 (chr. 8 q24.13) and the cohort mean *MYC* level.

Supplementary References

1. Rhodes DR, Yu J, Shanker K, Deshpande N, Varambally R, Ghosh D, et al. ONCOMINE: a cancer microarray database and integrated data-mining platform. Neoplasia 2004; 6(1): 1-6
2. Mizuno H, Kitada K, Nakai K, Sarai A. PrognoScan: a new database for meta-analysis of the prognostic value of genes. BMC Med Genomics 2009; 2:18
3. Uhlén M, Björling E, Agaton C, Szigyarto CA-K, Amini B, Andersen E, Andersson AC, Angelidou P, Asplund A, Asplund C, et al. A human protein atlas for normal and cancer tissues based on antibody proteomics. Mol Cell Proteomics 2005: 4:1920–1932
4. Choi W, Porten S, Kim S, Willis D, Plimack ER, Hoffman-Censits J, Roth B, Cheng T, Tran M, Lee IL, et al. Identification of distinct basal and luminal subtypes of muscle-invasive bladder cancer with different sensitivities to frontline chemotherapy. Cancer Cell 2014; 25(2): 152-65
5. Cheng SH, Huang TT, Cheng YH, Tan TBK et al. Validation of the 18-gene classifier as a prognostic biomarker of distant metastasis in breast cancer. PLoS One 2017; 12(9): e0184372
6. Lee YY, Kim TJ, Kim JY, Choi CH et al. Genetic profiling to predict recurrence of early cervical cancer. Gynecol Oncol 2013; 131(3): 650-4
7. Gravendeel LA, Kouwenhoven MC, Gevaert O, de Rooi JJ et al. Intrinsic gene expression profiles of gliomas are a Worse predictor of survival than histology. Cancer Res 2009; 69(23): 9065-72
8. Woo HG, Lee JH, Yoon JH, Kim CY et al. Identification of a cholangiocarcinoma-like gene expression trait in hepatocellular carcinoma. Cancer Res 2010; 70(8): 3034-41
9. Landi MT, Dracheva T, Rotunno M, Figueroa JD et al. Gene expression signature of cigarette smoking and its role in lung adenocarcinoma development and survival. PLoS One 2008; 3(2): e1651
10. Cirenajwis H, Ekedahl H, Lauss M, Harbst K et al. Molecular stratification of metastatic melanoma using gene expression profiling: Prediction of survival outcome and benefit from molecular targeted therapy. Oncotarget 2015; 6(14): 12297-309
11. Pérez-Mancera PA, Rust AG, van der Weyden L, Kristiansen G et al. The deubiquitinase USP9X suppresses pancreatic ductal adenocarcinoma. Nature 2012; 486(7402): 266-70
12. Filion C, Motoi T, Olshen AB, Lae M, Emnett RJ, Gutmann DH, Perry A, Ladanyis M, Labelle Y. The EWSR1/NR4A3 fusion protein of extraskeletal myxoid chondrosarcoma activates the PPARG nuclear receptor gene. J Pathol. 2009; 217(1): 83–93
13. Kim J, Woo AJ, Chu J, Snow JW, Fujiwara Y, Kim CG, et al. A Myc network accounts for similarities between embryonic stem and cancer cell transcription programs. Cell 2010; 143(2): 313-24
14. Müller F-J, Laurent LC, Kostka D, Ulitsky I, Williams R, Lu C, et al. Regulatory networks define phenotypic classes of human stem cell lines. Nature 2008; 455(7211): 401-5
